# Decoding Parametric Grip‐Force Anticipation From fMRI Data

**DOI:** 10.1002/hbm.70154

**Published:** 2025-02-12

**Authors:** Guido Caccialupi, Timo Torsten Schmidt, Till Nierhaus, Sara Wesolek, Marlon Esmeyer, Felix Blankenburg

**Affiliations:** ^1^ Neurocomputation and Neuroimaging Unit (NNU), Freie Universität Berlin Berlin Germany; ^2^ Berlin School of Mind and Brain, Humboldt Universität zu Berlin Berlin Germany

**Keywords:** action selection, fMRI, grip‐force, motor planning, MVPA, prospective working memory, working memory

## Abstract

Previous functional magnetic resonance imaging (fMRI) studies have shown that activity in premotor and parietal brain‐regions covaries with the intensity of upcoming grip‐force. However, it remains unclear how information about the intended grip‐force intensity is initially represented and subsequently transformed into a motor code before motor execution. In this fMRI study, we used multivoxel pattern analysis (MVPA) to decode where and when information about grip‐force intensities is parametrically coded in the brain. Human participants performed a delayed grip‐force task in which one of four cued levels of grip‐force intensity had to be maintained in working memory (WM) during a 9‐s delay‐period preceding motor execution. Using time‐resolved MVPA with a searchlight approach and support vector regression, we tested which brain regions exhibit multivariate WM codes of anticipated grip‐force intensities. During the early delay period, we observed above‐chance decoding in the ventromedial prefrontal cortex (vmPFC). During the late delay period, we found a network of action‐specific brain regions, including the bilateral intraparietal sulcus (IPS), left dorsal premotor cortex (l‐PMd), and supplementary motor areas. Additionally, cross‐regression decoding was employed to test for temporal generalization of activation patterns between early and late delay periods with those during cue presentation and motor execution. Cross‐regression decoding indicated temporal generalization to the cue period in the vmPFC and to motor‐execution in the l‐IPS and l‐PMd. Together, these findings suggest that the WM representation of grip‐force intensities undergoes a transformation where the vmPFC encodes information about the intended grip‐force, which is subsequently converted into a motor code in the l‐IPS and l‐PMd before execution.

## Graphical abstract

In a functional magnetic resonance imaging study, we employed a delayed grip‐force task and time‐resolved MVPA to decode where and when information about grip‐force intensities is parametrically coded. With a combination of support vector regression and cross‐regression decoding, we tested the translational processes between early and late delay periods and their similarity to action‐selection and motor execution. During the early delay period, we found the ventromedial prefrontal cortex to code the intended grip‐force intensity. This representation is then translated into motor‐like action‐specific codes during the late delay period, involving the intraparietal sulcus, left dorsal premotor cortex, and supplementary motor areas.

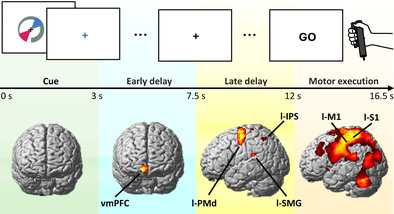

## 1 Introduction

Imagine a mountain bike rider steering down a challenging section of a gravel hill slope. As they recognize an impending transition to steeper and firmer terrain, they decide to reduce the bicycle's speed by applying a grip‐force to the handlebars. However, to avoid a severe fall on the unstable terrain, the bike rider must prepare and delay the grip‐force application. This scenario illustrates the continuity of serial translational processes, from the selection of an intended action to the motor planning of a grip force application through working memory (WM) maintenance, to motor execution. Until today, there has been substantial research on how specific actions are selected (Soon et al. 2008, 2013; Bode and Haynes 2009). However, it remains challenging to thoroughly characterise the translational processes from action selection to motor execution, in particular when an abstract goal is transformed into a motor movement plan (Kim, Avraham, and Ivry 2021). Therefore, systematically testing where in the human brain an intended action is represented during an early phase (after action selection) as compared to a later period (closer to motor execution) can provide further insight into this process.

The application of decoding techniques to functional magnetic resonance imaging (fMRI) data have provided a powerful tool for investigating where information is represented in the human brain (Haynes and Rees 2006; Norman et al. 2006). In particular, the use of multivoxel pattern analysis (MVPA) in delayed response paradigms (e.g., Harrison and Tong 2009) has proven successful for testing where and when information is encoded during different processing phases of WM (Schmidt, Wu, and Blankenburg 2017; Hebart et al. 2018). Of particular interest in WM studies are the transformation phases from the initial presentation of a stimulus (or task cue) to its representation during the early and late phases of a delay period. Previous research has shown that stimulus information is represented in sensory areas during and immediately after the stimulus presentation in visual (Christophel et al. 2015), tactile (Schmidt et al. 2021), and auditory (Uluç et al. 2018) WM studies. These studies further describe how information is transformed into more abstract representations in higher‐order sensory and multimodal cortices depending on task demands (Christophel et al. 2017). While these studies focus on the WM delay period, they do not examine how this information is translated into a motor plan for execution (Bode and Haynes 2009). In more ecologically valid scenarios, however, the primary function of WM is assumed to be using information to act on the environment (Parr and Friston 2017). As a result, recent research has emphasized the importance of studying how WM information is transformed into specific action plans (e.g., Van Ede and Nobre 2023). To date, only a few studies have investigated how information is encoded when a specific motor plan is cued, maintained, and then executed after a delay period (e.g., Soon et al. 2008, 2013; Bode and Haynes 2009; Gallivan, Johnsrude, and Flanagan 2015; Van Ede 2020; Boettcher et al. 2021; Ariani, Shahbazi, and Diedrichsen 2024).

Recent fMRI MVPA studies have shown that action‐specific information, such as the outcome of an intended action (or its “goal”; Hamilton and Grafton 2008; Cattaneo et al. 2009; Turella, Rumiati, and Lingnau 2020), can be decoded from the ventromedial prefrontal cortex (vmPFC) during an early delay (ED) period, even before motor planning begins (Soon et al. 2008, 2013; Ruiz et al. 2024). This finding aligns with a two‐stage model in which information about the intended action is first selected, and then, motor movements are specified and prepared for execution (Boettcher et al. 2021). This notion aligns with findings from transcranial magnetic stimulation (TMS) and fMRI decoding studies (Tecilla et al. 2022), which suggest a distinction between a motor decision phase in which a motor plan is selected from alternative actions, and a subsequent movement planning phase (Cisek and Kalaska 2005; Nakayama et al. 2008; Tecilla et al. 2022; Ruiz et al. 2024), with distinct brain regions being recruited during each phase. The above‐chance decoding in the vmPFC is also consistent with findings from a recent EEG study (Boettcher et al. 2021), which showed that in the absence of sensory stimulation, the electrophysiological brain activity reflects the outcome of an intended action before the instruction of movements (Ruiz et al. 2024) or selection of motor parameters (e.g., specific muscle fibres; Mizuguchi et al. 2011, 2013; Mizuguchi, Nakata, and Kanosue 2014; Ali, Montani, and Cesari 2023). Collectively, these studies highlight a distinction between the initial motor‐decision stage and the subsequent planning stage, emphasizing that motor planning does not commence until the intended action is selected.

After action selection, motor planning occurs (Nakayama et al. 2008; Tecilla et al. 2022; Ruiz et al. 2024) and is conceived as a neural process that optimizes an ideal preparatory state for motor output generation (Vyas et al. 2020; Churchland et al. 2010; Shenoy, Sahani, and Churchland 2013; Ariani, Pruszynski, and Diedrichsen 2022). Animal studies on motor planning, which employed single‐cell recordings, have consistently found preparatory signals in patterns of neuronal firing in the dorsal premotor cortex (PMd; e.g., Cisek and Kalaska 2004, 2010; Hoshi and Tanji 2006, 2007), the supplementary motor area (SMA; e.g., Hoshi and Tanji 2004a; Hoshi and Tanji 2004b), and the PPC (e.g., Cui and Andersen 2007; Cui and Andersen 2011; Andersen and Cui 2009). Human fMRI MVPA studies have shown that activity in parieto‐frontal brain regions during the planning of grasping actions was predictive of several movement properties, such as the kinematics of grip types (Gallivan et al. 2011b; Ariani, Wurm, and Lingnau 2015; Ruiz et al. 2024), the action order (Yokoi and Diedrichsen 2019; Ariani et al. 2021; Ariani, Pruszynski, and Diedrichsen 2022), and the to‐be utilized effector (Gallivan et al. 2011a, 2013; Leoné et al. 2014; Turella, Rumiati, and Lingnau 2020). Delayed grip‐force tasks have also been used to investigate the neuronal underpinnings of the anticipatory scaling of grip‐force intensities (Cole and Rotella 2002; Chouinard, Leonard, and Paus 2005; Nowak et al. 2009; Van Nuenen et al. 2012). In particular, fMRI studies using univariate analyses have shown that the strength of the blood‐oxygen‐level‐dependent (BOLD) signal in the PMd and PPC reflects the anticipated grip‐force intensity (Van Nuenen et al. 2012; Mizuguchi, Nakata, and Kanosue 2014).

To isolate neural correlates of motor planning from execution, previous studies have used delayed‐movement tasks, which resemble the delayed‐match‐to‐sample (DMTS) or categorization tasks commonly used in WM research. These include delayed grip‐force tasks (e.g., Van Nuenen et al. 2012) that, requiring the maintenance of a graded motor parameter over time, are highly similar to parametric WM tasks (e.g., Romo et al. 1999; Brody 2003). Romo and co‐workers revealed that maintained continuous sensory parameters, such as vibrotactile stimulation intensities, are encoded in persistent, monotonically tuned neural activity in the (right) inferior prefrontal cortex (e.g., Romo et al. 1999; Brody 2003). More recently, MVPA has been essential in showing that premotor and parietal brain regions can parametrically represent and maintain information in distributed patterns of brain activity (Schmidt, Wu, and Blankenburg 2017; Uluç et al. 2018; Wu et al. 2018; Uluç et al. 2020). However, it remains to be tested whether parametric information about grip‐force intensities is also encoded in multivariate patterns during action selection and motor planning (i.e., during the early and late delay periods).

The present study investigates whether, where and when grip‐force intensities can be parametrically decoded from fMRI data in a delayed grip‐force task. To relate our findings to the parametric WM findings, we modified the established DMTS to a delayed grip‐force task. However, instead of maintaining sensory properties of stimuli such as vibrotactile frequencies (e.g., Romo et al. 1999; Schmidt, Wu, and Blankenburg 2017), participants had to anticipate and maintain grip‐force intensities before execution. As shown in previous fMRI studies, the grip force is a motor component that can be easily maintained during a delay period and comprises a mental representation of a content with high ecological validity (van Nuenen et al. 2012; Mizuguchi et al. 2013). Moreover, it can be carefully controlled in an experimental setting and be studied in isolation from other motor components (e.g., finger movement kinematic). Beyond these, the neural correlates of grip force are currently researched as proxy for assessing human health. For instance, beta cortico‐muscular coherence has been reported to be reduced in individuals with Parkinson's disease (Zokaei et al. 2021). Taken together, testing the neural correlates of parametrically modulated grip‐force intensities provides good experimental control and has potential for translational research approaches.

In the study at hand, we decoded parametric grip‐force intensities from the early (ED) and late delay (LD) periods using time‐resolved MVPA (by employing support vector regression [SVR]). In addition, cross‐regression decoding was used to test whether neural representations during the ED and LD periods were similar to those during the motor execution and cue periods (Meyers et al. 2008; King and Dehaene 2014; Hebart and Baker 2018; Hebart et al. 2018; Ariani, Wurm, and Lingnau 2015; Ariani, Oosterhof, and Lingnau 2018; Nierhaus et al. 2023). Based on the literature, we hypothesized that parametric neural representations of grip‐force intensities can be identified in action‐specific brain regions, such as the PMd, SMA, and PPC, and that these codes differ from early representations in the vmPFC.

## 2 Materials and Methods

### 2.1 Participants

All participants (*N* = 33) were healthy and right‐handed, as assessed by the *Edinburgh Handedness Inventory* (Oldfield 1971). Before the experiment, they provided written informed consent to participate in the study in accordance with protocols approved by the local ethics committee of *Freie Universität Berlin* (003/2021, Berlin, Germany).

To account for potential biases and confounds, only *N* = 22 participants were included in the analysis (age: 30 ± 6.31, 4 female). Five participants were excluded due to low performance (i.e., percentage of trials with accurate responses < 25% in at least one experimental run and one experimental condition). Another six participants were excluded due to frequent grip‐force application during the delay period (i.e., percentage of trials with applied force > 75% in at least one run and one condition). To further ensure that the analyses were not confounded by early motor responses, we excluded from the dataset all trials in which any grip‐force was applied during the delay period (cutoff: mean grip‐force ≥ 0.05 on a scale from 0 to 1).

### 2.2 Experimental Procedure

**FIGURE 1:**
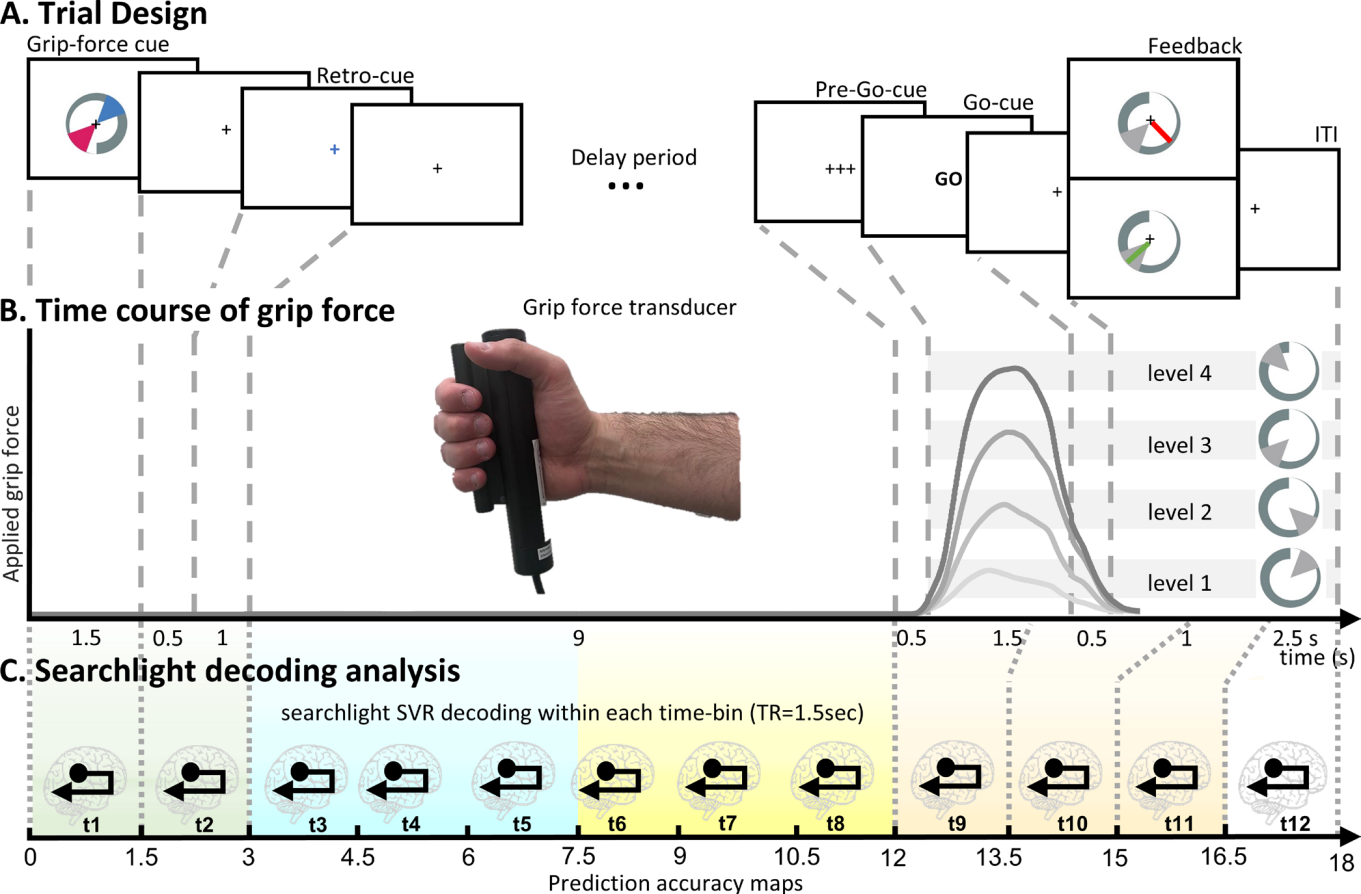
(A) *Delayed grip‐force paradigm*. Two grip‐force levels were presented on a *grip‐force cue* (i.e., a grey circular increasing bar with one level represented in cyan and the other in red, as sectors of 50° on the *indicator*). A *visual retro‐cue* (i.e., a cyan or red cross displaced in the centre of the screen) indicated which grip‐force had to be anticipated and maintained during a subsequent 9‐s *delay period*. After this *delay*, participants prepared and performed the grip force upon the presentation of a *pre‐Go* (0.5 s) and a *Go‐cue* (1.5 s). Subjects responded with their right hand by differently squeezing a grip‐force device, and feedback representing the applied force was provided 1.5 s after the *Go‐cue*. (B) Different shades of grey represent time courses of mean grip forces applied during the experimental‐trial, calculated over the accurate trials per condition (i.e., per grip‐force level). Light grey bars displayed in the background represent grip‐force levels. (C) Represents the MVPA with a searchlight and a time‐resolved approach adopted across four time periods of the trial. The pastel green background represents a 3‐s cue period, the light cyan background depicts a 4.5‐s early delay period, the light yellow background refers to a 4.5‐s late delay period, and the light red background marks a 4.5‐s motor execution period.

After participants entered the MRI scanner, the grip‐force transducer was calibrated to participants' maximum grip‐force. Next, participants were introduced to the four grip‐force levels that served as the target grip‐force intensities in the main experiment. In this training phase, they were familiarized with the grip‐force transducer by performing 24 trials with real‐time visual feedback of the applied grip‐force. In 24 additional trials, they were trained on the experimental task (see below) during the acquisition of a structural MRI scan. Finally, participants performed the delayed grip‐force task (illustrated in Figure 1A) in four runs during fMRI scanning.

### 2.3 Grip‐Force Assessment and Stimuli

Based on the visual presentation of a *grip‐force cue* and a *retro‐cue*, participants had to apply a grip force via the right hand on a cylindrical MR‐compatible grip‐force transducer (i.e., a *force fibre optic response pad*, Current Designs, HHSC‐1 × 1‐GRFC‐V2; illustrated in Figure 1B). Grip‐force intensity was sampled at a 4000 Hz rate throughout the task (and downsampled by a factor of one‐third for analyses) to ensure that participants only applied force during the execution period of the trials, allowing the exclusion of trials where participants applied force during other periods of the experimental trials.

**FIGURE 2:**
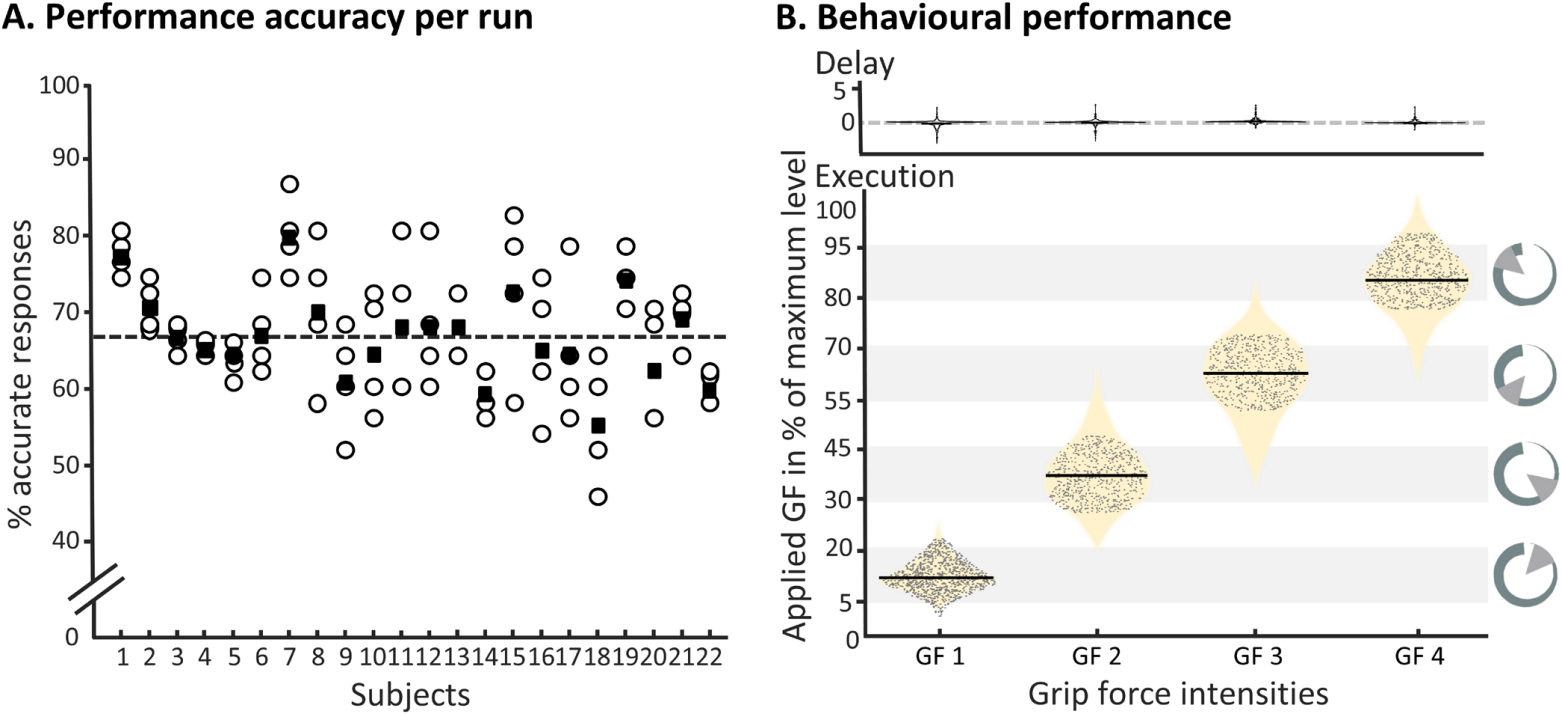
Behavioural assessment. (A) Shows that participants performed consistently across all four runs of the delayed grip‐force task. Circles represent the performance of accurate grip‐force application in each of the four runs (for each participant); filled squares represent the mean performance across runs. The overall mean performance across participants is represented by the dashed black line. (B) Displays the grip‐forces applied during the delay period (upper display) and the execution period (lower displays). We made sure that only trials were included in the analysis, where participants did not press the grip‐force device during the delay period (upper panel), and accurate grip‐force was considered within the target level ± 20° (lower panel). Light grey bars in the background represent the cued grip‐force (GF) levels. For each of the four cued GF levels a violin plot (beige) illustrates the distributions of applied grip‐forces, with the trials included in the analysis indicated by black dots.

The visual *grip‐force cue* was presented in the centre of the screen, utilizing *Psychtoolbox‐3* (Brainard 1997). The visual cues were composed of a 360° circular display increasing in thickness, corresponding to an increase in grip‐force where the maximum corresponded to 75% of the individual maximum grip‐force. Four grip‐force levels were indicated by the display of a range, defining a sector of 50° of the grip‐force spectrum, with grip‐force level 1: 20°–70° (5%–20% of maximum grip force level), level 2: 110°–160° (30%–45%), level 3: 200°–250° (55%–70%), and level 4: 290°–340°(80%–95%) (as illustrated in Figure 1B, right side; and Figure 2B). The *grip‐force cue* was presented for each trial with a random degree of rotation (as illustrated in Figure 1A). This complex presentation of the cue was chosen to render the sensory and motor information orthogonal, thus making it unlikely that participants could solve the task by maintaining the sensory information without directly transforming it into a grip‐force intensity. It further prevents the possibility of decoding residues of any (parametric) visual stimulus feature (e.g., length) during the delay phase.

### 2.4 Experimental Task

During fMRI scanning, participants performed a delayed grip‐force task. Each trial started with the presentation of a *grip‐force cue*, comprised of two grip‐force levels presented in cyan and red (illustrated in Figure 1A). A *retro‐cue* indicated which of the two grip‐force intensities had to be maintained and executed. The combinations of displayed grip‐force levels were balanced so that all combinations of two different levels were presented equally often. After a *delay period* of 9 s, participants had to apply the anticipated grip‐force upon the display of a *Go‐cue*; importantly, in the absence of the grip‐force cue. A *pre‐Go cue* was presented for 0.5 s before the response to allow optimal response timing during the 1.5 s response window. Finally, visual *feedback* was provided by showing the applied force on a screen as a green radian if accurate, or as a red radian if inaccurate, that is, outside of the cued grip‐force level (compare Figure 1A). The mean grip‐force during the last 0.75 s of the response window was evaluated to assess accuracy of responses. For the analysis, trials were considered accurate when the applied force was within the target range ±20° (5% of maximum grip force).

Four experimental runs comprised 48 experimental trials each, supplemented with 12 catch trials with a shorter delay period of 6.0 s, 4.5 s, 3.0 s, or 1.5 s. Catch‐trials were designed to enhance participants' readiness to prepare and execute the grip‐force task, and thus the continuous maintenance of the force intensity. Catch trials were not included in the analyses. Trial order was fully randomized within a run, where each experimental condition (i.e., the cueing of each of the four to‐be anticipated grip‐force intensities) was presented equally often (i.e., 12 times per run).

### 2.5 fMRI Data Acquisition and Preprocessing

fMRI data were acquired in four runs of 18 min and 40 s each on a 3 T Siemens Prisma at the *Center for Cognitive Neuroscience Berlin* of the *Freie Universität Berlin*. For each run, 745 functional images were obtained with an EPI sequence (64‐channel head coil, 48 slices, interleaved order; TR = 1.5 s, 2.5 × 2.5 × 2.5 mm voxel size; multiband acquisition with acceleration factor of 3).

Trial onsets were time‐locked to the functional image acquisition to allow a time‐resolved analysis (see below). fMRI data processing was performed using SPM12, r7771 (*Wellcome Trust Centre for Neuroimaging*, *Institute for Neurology*, *University College London*). To preserve the spatiotemporal structure of the fMRI data, preprocessing was limited to motion correction by spatial realignment to the runs mean image, as implemented in SPM12.

#### 2.5.1 Time‐Resolved Searchlight Decoding of Grip‐Force Anticipation

We applied an MVPA searchlight approach to identify brain regions that exhibited multivariate parametric codes of grip‐force anticipation during the delay period. Finite impulse response (FIR) models were used to obtain run‐wise beta estimates for each 1.5 s time‐bin of the trials. Then, 12 consecutive time‐bins were modelled (as illustrated in Figure 1C), comprising 6 time‐bins throughout the delay period, 2 additional time‐bins before the delay period, and 4 time‐bins from grip‐force execution. Temporal filtering was implemented within the first‐level general linear model (GLM) using a high‐pass filter with a cutoff of 128 Hz. The first‐level model included 220 regressors (4 conditions × 12 time‐bins × 4 runs complemented with the 6 realignment parameters). To balance the number of trials included in each condition, allowing equally reliable beta‐estimates, we subsampled the trial number to *N* = 5 for condition (including the trials with most precise grip‐force performance).

To identify where and when information about the maintained grip‐force was encoded in the brain, we applied time‐resolved MVPA using SVR (Kahnt et al. 2011), which can be considered a multivariate pendant of a parametric coding, a method previously applied in a series of WM decoding experiments (e.g., Christophel, Hebart, and Haynes 2012; Schmidt, Wu, and Blankenburg 2017; Uluç et al. 2020; Pennock et al. 2021). Applying a searchlight approach (*r* = 4 voxel) independently for every time‐bin allows testing for local multivariate representations (i.e., activation patterns of voxels) that code parametric grip‐force anticipations. An SVR model is trained to predict grip‐force (i.e., the four grip‐force intensities) based on a multivariate data vector (i.e., multivoxel activation pattern). This is similar to a univariate regression approach, where prediction of the dependent variable is however solely based on one independent variable (see Kahnt et al. 2011 for more information).

To account for differences in the difficulty of grip‐force performance, the distances between the four SVR labels were adjusted using Fechner's law (Fechner 1948). First, we accounted for differently perceived difficulties by log‐transforming the applied grip‐force intensities, which were grouped in four normal distributions of applied grip‐force. After, we calculated the three distances between the four labels of the SVR ($d_{i},d_{i+1},d_{i+2}$). Specifically, such distances were obtained by calculating the inverse of the difference between the means for each combination of increasing grip‐force intensities ($d_{i}=\frac{1}{|\mu_{i}-\mu_{i}+1|}$). We used the resulting distances to adjust the SVR's labels (with resulting labels: −1.5, −0.58, 1.36, and 4.33).

Beta estimates of each condition were first normalized (i.e., z‐scaled) across the samples for each voxel as implemented in the Decoding Toolbox (TDT; Hebart, Görgen, and Haynes 2015) and forwarded to a fourfold leave‐one‐run‐out cross validation schema. To make our data comparable to previous reports, we used the prediction accuracy as a measure, defined as the Fisher's z‐transformed correlation coefficient between the predicted value levels and the actual value levels of the test dataset (in TDT: “zcorr,” see also Kahnt et al. 2011). The centre of the searchlight was moved voxel wise through the brain, and prediction accuracy values were saved as corresponding whole‐brain accuracy maps. In this way, we obtained an accuracy map for every subject and time‐bin, reflecting local activation patterns that code grip‐force intensity in a multivariate way.

Prediction accuracy maps were entered into a second‐level analysis to test for above‐chance decoding in terms of SPM's flexible factorial design implementation of an ANOVA. We used t‐contrast to test for above‐chance decoding across different time‐bins. All results are reported at a threshold of *p* < 0.05 family‐wise error (FWE) corrected at the voxel level.

#### 2.5.2 Control Analyses: Decoding the Non‐Memorized Grip‐Force, Label Permutation Control Analysis, and HRF‐Convolved GLM Univariate Analysis

To test for the specificity of the main analysis, we performed a second MVPA as a control analysis. Namely, we tested for above‐chance prediction accuracy for the non‐maintained grip‐force intensities. A new FIR model was estimated, in which four sets of FIR regressors modelled those trials in which a grip‐force level was presented and not retro‐cued. As previously done in the main analysis, 12 consecutive time‐bins were modelled for the 5 most accurate trials of each condition. High‐pass filtered data (cut‐off 128 s) were included in a corresponding first‐level GLM model with 220 regressors (4 conditions × 12 time‐bins × 4 runs complemented with the 6 realignment parameters). Therefore, each beta image was estimated with equal amounts of data (i.e., modelling the same number of trials) as in the main analysis. Beta images were entered into an identical SVR searchlight and second‐level analysis as in the main analysis.

Additionally, to control whether prediction accuracies of the main analysis were based on the parametric coding of grip‐force intensity anticipation, we performed a label‐permutation test. Here, the labels of the conditions entered into the SVR were permuted. As previously applied (see Schmidt, Wu, and Blankenburg 2017; Uluç et al. 2020), higher distance of the labelling from the original order should result in reduced decoding accuracies (getting to zero for the highest distance, i.e., completely unordered labelling). For all possible permutations, the distance from the rank order was calculated as the sum of the absolute difference of adjacent ranks (e.g., the linear order of grip‐force level 1, 2, 3, 4 has a distance of ranks of sum (|1 − 2| + |2 − 3| + |3 − 4|) = 3 and the permuted labelling 2, 1, 3, 4 corresponds to the sum (|2 − 1| + |1 − 3| + |3 − 4|) = 4, resulting in a difference of 1 from the linear order). Thereby, the permutation analysis congregated permutations into four classes of distances from the linear order. For all permutations of labels, the same SVR whole‐brain searchlight analysis as in the main decoding was carried out.

Finally, we explored univariate activation differences during the delay period. We performed a univariate control analysis to test for parametric increases of activity during the delay period, that is, an analysis based on classic HRF convolved GLM approach. To this end, functional images were coregistered, normalized and spatially smoothed (with SPMs default of 8 mm FWHM). On the first level, the following regressors were modelled as HRF‐convolved boxcar functions for the given phases of the trials: the cue period (i.e., t1–t2), delay period (i.e., t3–t8) + parametric modulator, motor execution period (i.e., t9–t11) + parametric modulator, and the motion parameters as regressors of no interest. “Fechner‐corrected” labels (see previous section) were used to weight parametric modulators. We used one sample *t* test to assess group level effects of first‐level contrasts.

#### 2.5.3 Test for Temporal Generalization: Cross‐Regression Decoding

To test whether the multi‐voxel activation patterns in the ED and LD periods were similar to those in the cue and motor execution periods, we conducted a cross‐regression decoding analysis (as test for temporal generalization). We use the term “cross‐regression” instead of “cross‐classification” because a linear SVR model was trained to predict a continuous variable (i.e., grip‐force intensity) rather than to classify into a category (Kahnt et al. 2011; Schmidt, Wu, and Blankenburg 2017). Nonetheless, the logic underlying the use of cross‐classification to test for temporal generalization applies to SVR approaches as well (Bode et al. 2022). Similar to previous MVPA studies (Meyers et al. 2008; King and Dehaene 2014; Hebart et al. 2018), we tested for temporal generalization by training on each time‐bin and, respectively, testing on all other time‐bins. The cross‐regression analysis was based on the same data as the main analysis, namely: the beta estimates derived from the same FIR model were used. SVRs was trained and tested on all time bins (t1–t12 × t1–t12) using four‐fold leave‐one‐run‐out cross‐validation as in the main analysis, resulting in 144 cross‐regression accuracy maps.

For the statistical assessment, we averaged prediction accuracy maps to fall into 16 cross‐regression accuracy maps to reflect the 4 time periods investigated in the main decoding analysis, that is, the cue period, ED period, LD period, and motor execution period. Averaging was performed using the ImCalc tool as implemented in SPM. After normalization and smoothing, the 16 cross‐regression prediction accuracy maps of 22 participants were entered into a second‐level analysis to test for above‐chance decoding using SPM's flexible factorial design specification of an ANOVA. This design included one factor with four levels for the training periods, and a second factor with four levels for the testing periods. We used t‐contrasts to assess above‐chance cross‐regression decoding on each of the 16 maps. All results are reported at *p* < 0.05 FWE corrected at the voxel level.

## 3 Results

### 3.1 Behavioural Performance

The participants included in the main analysis (*N* = 22) responded with accurate application of grip‐force (i.e., they were in the target range ± 20°) in 67% ± 5.5% (mean ± SD) of the trials. Average performance across runs indicates small training effects over time: run 1: 66% ± 7.7%; run 2: 64% ± 7%; run 3: 70% ± 10%; run 4: 70% ± 7% (performance per participant and run are illustrated in Figure 2A). When tested with a 1 × 4 repeated‐measures ANOVA, this effect of runs was significant (*df* = 3, F = 2.73, *p* = 0.0491, *η* ^2^ = 0. 0178), while post hoc *t* tests did not reach significance after Bonferroni correction. These findings render major learning effects on the neuroimaging results rather unlikely.

To test for performance differences across the four to‐be‐applied grip‐force intensities, we performed a 1 × 4 repeated‐measures ANOVA, which was significant (*df* = 3, *F* = 78.35, *p* = 0.001, *η* ^2^ = 1. 8017). This was also reflected by significant post hoc *t* tests, which revealed that the application of grip‐force intensity 1 was easier than the other grip‐force intensities. After Bonferroni correction, the mean of the performance accuracy for grip‐force intensity 1 was significantly greater than intensity 2 (M_diff_ = 26%, *p* = 0.001), intensity 3 (M_diff_ = 31%, *p* = 0.001), and intensity 4 (M_diff_ = 26%, *p* = 0.001). Violin plots in Figure 2B display the distribution of responses (i.e., accurate and non‐accurate performances) in terms of applied force on the grip‐force device for the respective trial types (i.e., grip‐force intensities); the data points display only accurate responses. We further ensured that the included participants did not apply force during the *delay period* (see Figure 2B, upper display). These results are in line with the expected and previously reported Fechner's Law effect for perceived difficulties of to‐be executed force intensities (Jones 1989). Therefore, we applied a correction to the fMRI data modelling (see Section 2).

### 3.2 Multivariate Mapping of Regions That Code Grip‐Force Anticipation

**FIGURE 3:**
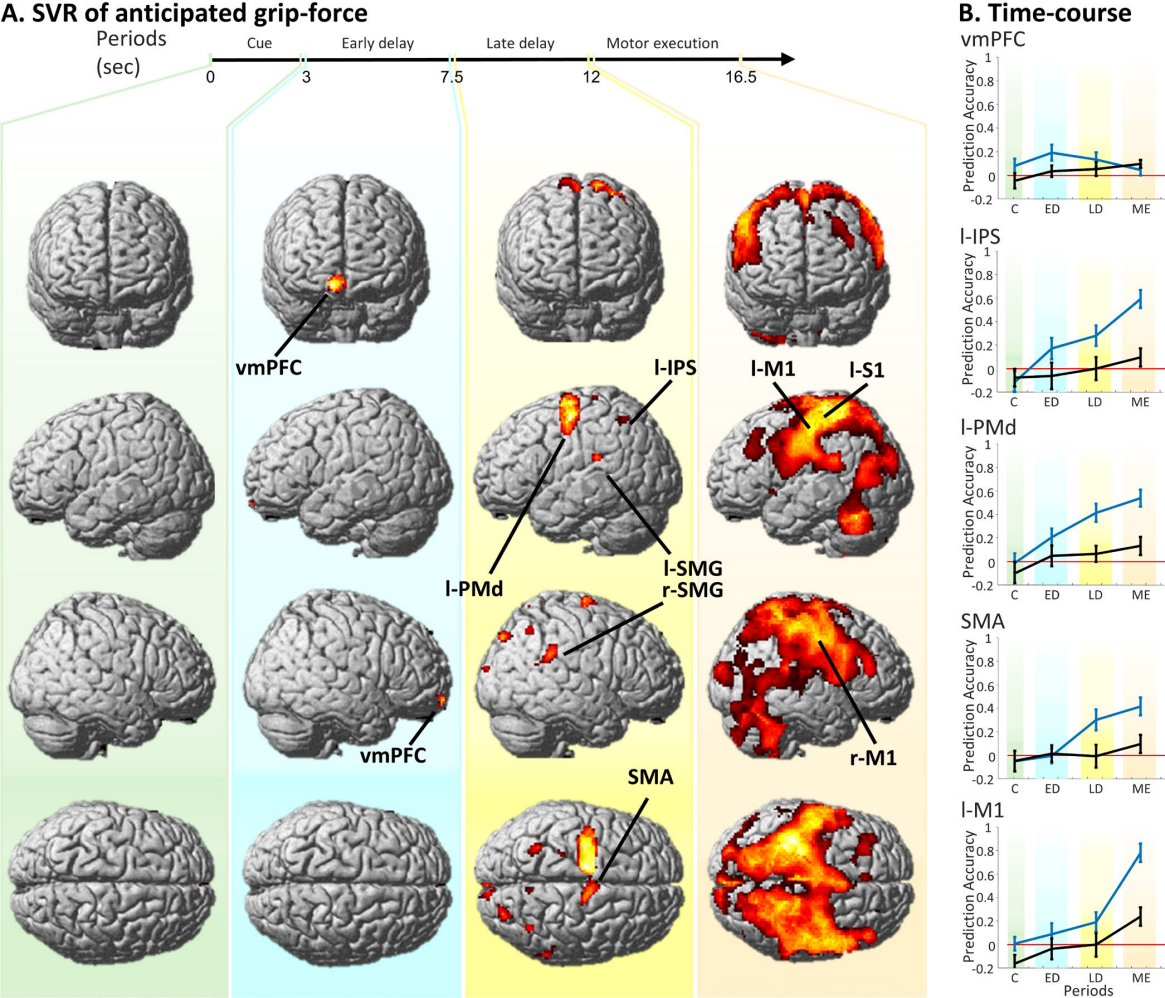
Results of time resolved support vector regression analysis. A. Displays brain regions that parametrically code grip‐force intensities during the cue period (C), early delay period (ED), late delay period (LD), and motor execution period (ME). Brain regions with above‐chance prediction accuracy are revealed by four t‐contrasts displayed at *p* < 0.05, FWE‐corrected. No brain region exhibited above‐chance decoding during C (as displayed in the first column, pastel green background). During the early delay period, a cluster in the vmPFC was revealed (second column, light cyan background). In the late delay period, a network including bilateral intraparietal sulcus (IPS), left dorsal premotor cortex (l‐PMd), and the supplementary motor area (SMA) was found (third column, light yellow background). Finally, a t‐contrast on the motor execution period revealed one extended cluster centred on the M1 and S1 (fourth column; light red background). B. Time courses of prediction accuracy values relative to the main decoding analysis (displayed in blue) and the control analysis (in black). Prediction accuracy values were extracted from the peak voxels of the five most representative clusters reported in Table 1. The time course represents four prediction accuracy values obtained by averaging prediction accuracy values of 12 time‐bins in correspondence of the four time periods (tested in the main analysis). As expected, prediction accuracy values for the control analysis do not overcame chance level. Please note that because time courses were extracted from the most significant voxels they are only displayed for descriptive purpose, and no further statistical testing was conducted to avoid circular conclusions.

To identify brain regions that encode parametric grip‐force anticipation, we conducted a time‐resolved whole‐brain SVR analysis. Within a second‐level ANOVA design, we computed t‐contrasts across prediction accuracy maps of 3 periods of interest, that is, the cue period (C, time‐bins t1–t2); the ED period (ED, t3–t5); and the LD period (LD, t6–t8); all *p* < 0.05 FWE‐corrected. No brain region exhibited above‐chance decoding during the cue period, only a small cluster in the vmPFC was revealed when assessed at *p* < 0.001 uncorrected. During the ED period, a cluster in the vmPFC was revealed. In the LD period, a network including bilateral intraparietal sulcus (IPS), left dorsal premotor cortex (l‐PMd), and the SMA was found (see Figure 3A, Table 1). Finally, a t‐contrast on the motor execution period (i.e., ME, t9–t11) revealed one extended cluster spanning bilateral primary motor cortex (M1) and primary somatosensory cortex (S1), parietal cortices, and the cerebellum (Figure 3A, fourth column, and Table 1).

**TABLE 1:** Regions that exhibit above‐chance prediction accuracy across the cue period, early and late delay periods and motor execution period, revealed by a t‐contrast displayed at *p* < 0.05, FWE corrected.

Cluster size	Anatomical region	Peak MNI coordinates			
		x	y	z	z‐Score
**Early delay period**					
231	Ventromedial PFC	6	60	−10	5.27
**Late delay period**					
1370	Left PMd	−26	−10	58	6.48
	SMA	−6	−6	46	5.16
156	Right SMG	52	−42	32	4.94
73	Left IPS	−30	−54	52	4.89
42	Right IPS	38	−58	48	4.74
100		26	−86	44	5.06
139		2	−96	14	4.95
156		52	−40	32	4.81
51		−56	−34	22	4.75
**Motor execution period**					
58,068	Left S1	−32	−34	58	11.42
	Left M1	−43	−14	48	8.46

To plot the temporal evolution of grip‐force specific decoding accuracy within the network of brain regions recruited during the ED and LD periods, we extracted the time course of prediction accuracies from the peak voxels of the identified clusters in the vmPFC, l‐IPS, l‐PMd, SMA and l‐M1 (see Table 1 for MNI coordinates). Time courses of mean prediction accuracies within the four time periods of the experimental trials are displayed in Figure 3A. As expected from the dynamics of the BOLD response and previous reports (e.g., Schmidt, Wu, and Blankenburg 2017; Uluç et al. 2020), the prediction accuracies peaked between 4 and 8 s after the start of the delay period (see also Supporting Information for time‐courses plotted for 12 time‐bins). The time course of the vmPFC displayed an early peak during the ED period, preceding those of action‐specific brain regions, which reached maximum in the LD period. All regions of interest, except the vmPFC, showed a substantial increase of prediction accuracy during the execution.

### 3.3 Control Analyses: Decoding Non Maintained Grip‐Force Intensities, Label Permutation Control Analysis, and HRF‐Convolved GLM Univariate Analysis

As a control analysis for the specificity of the main analysis, we tested if information on the non‐memorized (i.e., non‐cued) grip‐force intensity can be decoded. Therefore, we conducted an identical MVPA as the main analysis (based on FIR models that modelled the non‐cued intensity). Please note that the amount of cued and non‐cued grip‐force intensities was balanced within and across runs. The decoding analysis of the non‐maintained grip‐force intensity did not reveal any significant cluster (at *p* < 0.05 FWE‐corrected), corroborating the specificity of the main analysis. Time course data of this control analysis is displayed in Figure 3B and in the Supporting Information.

To further corroborating the specificity of the main analysis, we conducted label permutation testing and two second‐level analyses. First, we tested above‐chance decoding for the maximally permuted labels in terms of SPM's flexible factorial design implementation of an ANOVA. Convincingly, when t‐contrasts are computed on the ED and LD periods (as in the main analysis), we found no significant clusters throughout the whole brain with identical *p* < 0.05, FWE correction. Even when inspecting this contrast at *p* < 0.001 uncorrected. For illustrative purposes, we display the time courses of the label‐permutation tests with increasing dissimilarity to the original order for the peak voxels of the main analysis (see Figure S1A in the Supporting Information). As expected, the time course of the completely unordered labelling do not show above‐chance prediction accuracies throughout all phases of the experimental trials. As a second group‐level analysis, we entered prediction accuracy maps resulting from permutation labelling into a flexible factorial design with one factor for ordered and permuted levels (i.e., Ordered, Distance 1, Distance 2, Distance 3, and Distance 4), and a second factor for the 12 time‐bins. We computed parametric contrasts over the same time‐bins as in the main analysis, namely C, ED, LD, and ME, respectively. These contrasts corroborated the results of the main analysis, as they show across the time periods very similar clusters as the main analysis at *p* < 0.05 FWE corrected (see Figure S2A and Table S1).

In addition to the performed MVPA, we also explored univariate activation during the delay period. We used one sample *t*‐test to assess group level effects of first‐level contrasts. No significant parametric modulation was found during the delay phase (at *p* < 0.05 FWE‐corrected; cluster extent threshold > 10). In contrast, l‐M1 and right cerebellum demonstrated strong parametric activity during motor execution (see Figure S4A, and Table S2 in Supporting Information). These results make it unlikely that the parametric effects observed in the main MVPA analyses were mostly driven by univariate effects.

### 3.4 Testing for Temporal Generalization

**FIGURE 4:**
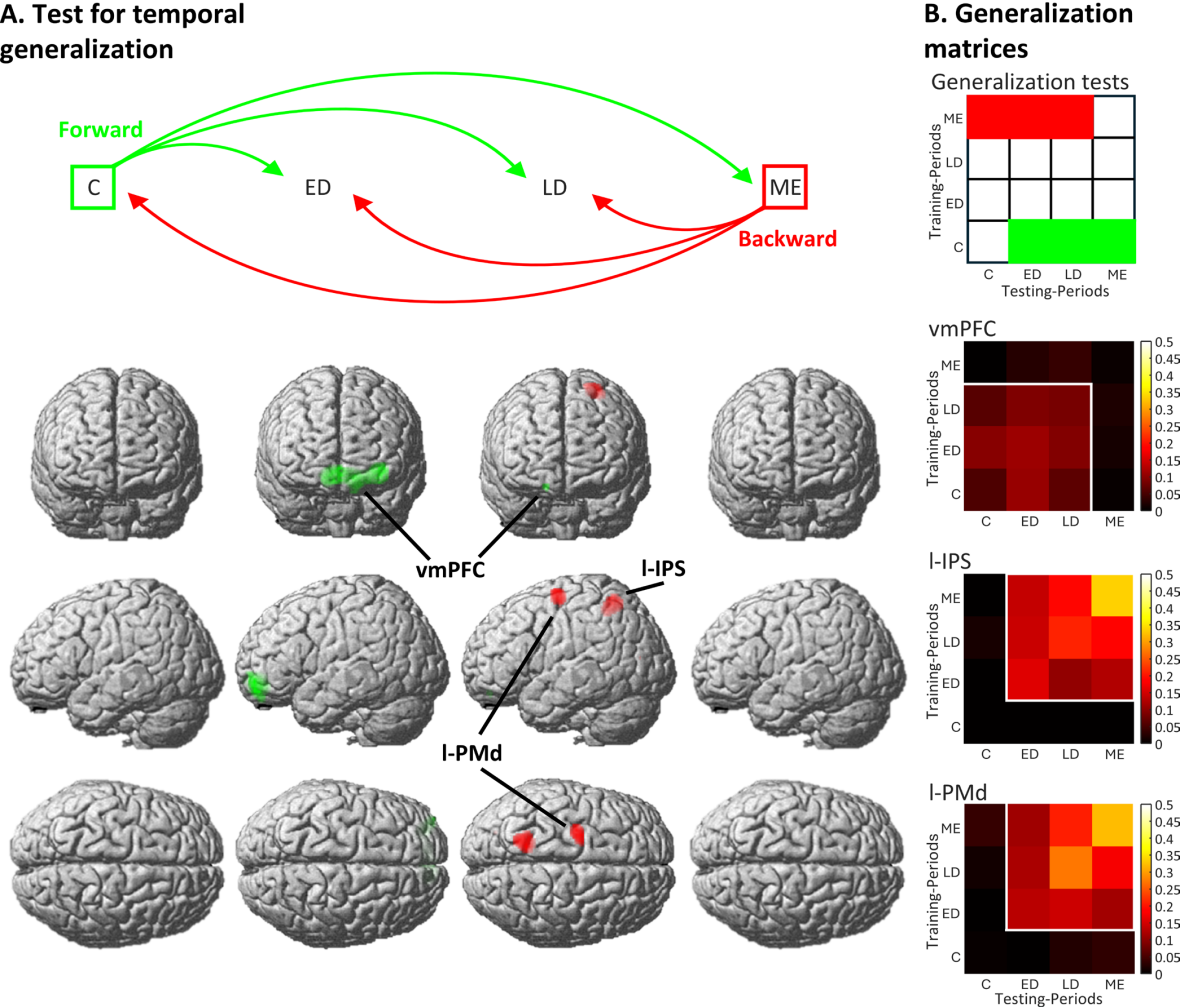
(A) The upper display shows four time periods: The cue period (C), early delay period (ED), late delay period (LD), and motor execution period (ME). The three green arrows illustrate forward generalization (where the SVR was trained on the C and tested, respectively, on the ED, LD, and ME); red arrows illustrate backward generalization (with training on the ME and testing on the LD, ED, and S). Six t‐contrasts were computed on the resulting cross‐regression accuracy, and results are showed in the lower display in four columns, corresponding to the testing periods. Results are coloured as forward (green) and backward (red) generalization. (B) The upper display indicates which fields of a generalization matrix correspond to forward and backward generalizations (as displayed in A). The generalization matrix represents testing periods on the *x‐axis* and training periods on the *y‐axis*. According to this schema, the lower display shows three temporal generalization matrices, each corresponding to a brain region that showed above‐chance decoding. These matrices display cross‐regression accuracy values for the peak‐activity voxel in the vmPFC, l‐IPS, and l‐PMd (as identified in the main decoding analysis). Sixteen cross‐regression accuracy values (reflecting the full range of tests across time‐periods; see Method section) are represented within each heatmap. Brighter colours represent higher cross‐regression accuracy values. The contours represent above‐chance cross‐regression decoding, as revealed by t‐contrasts on the peak voxel. Overall, the three generalization matrices indicate that prediction accuracy values are similar for both generalization directions (e.g., whether training is conducted on S and testing on ED, or vice versa). All brain regions exhibit relatively stable brain activity patterns between the two delay periods (i.e., when generalization tests are conducted between ED and LD).

To test whether multivariate patterns of grip‐force intensities found in the ED and LD periods were similar to those in the cue and motor execution periods, we conducted whole‐brain cross‐regression decoding analyses. All combinations of forward and backward generalization were tested (see Figure 4A). This means that in forward generalization all combinations of training on early time‐bins and testing on later time‐bins were computed to test if the WM representation is similar to an initially formed mental representation during the action selection. In contrast, backward generalization trained on the activation patterns during motor execution and tests on the preceding time‐bins to evaluate if during the WM period (i.e., anticipation of motor execution) already shows neuronal activation that is similar to the actual motor execution.

First, to test for forward generalization, we computed a t‐contrast across the accuracy maps from training on the cue period (C) and testing in the other periods. Results are displayed in Figure 4A in green (all results are displayed at *p* < 0.05 FWE‐corrected). Interestingly, a single cluster in the vmPFC was revealed, which is largely overlapping with the vmPFC of the main analysis. This cluster was found less pronounced in the LD period. Consistently, Figure 4B shows the stability of generalization when training on the ED period and testing on the LD phase for the vmPFC cluster. In contrast, no temporal generalization was found to motor execution even at *p* < 0.001, uncorrected, indicating that the initial mental representation might be transformed into a different code for motor execution.

Analogously, we tested for backward generalization by training SVRs on the motor execution period (ME) and testing on the preceding periods. Results are displayed in Figure 4A in red. Interestingly, only two clusters in the l‐PMd and l‐IPS were found, which largely overlap with those exhibited in the LD period of the main decoding analysis (compare Figure 3A). Also, this analysis did not reveal any temporal generalization to the cue period (even at *p* < 0.001, uncorrected). Thereby, forward and backward temporal generalization tests corroborate that over time the neural representation of the intended action is converted to a different mental representation. Early codes include the vmPFC, while later in the trial information is found in the l‐IPS and l‐PMd, indicating similar codes as during motor execution.

## 4 Discussion

In this fMRI study, we employed a delayed grip‐force task and time‐resolved MVPA to identify brain regions where information about grip‐force intensities is parametrically coded and transformed during the cue period, ED and LD periods, and motor execution.

As expected, information about the intended grip‐force intensity could only be decoded starting from the delay period (i.e., 3 s after start of cue presentation). In the ED period, we only found the vmPFC. Meanwhile, the LD period revealed action‐specific brain regions, including the l‐IPS, l‐PMd, and SMA. Thereby, our analysis suggests that the code with which a motor action is initially selected differs from how information is coded further towards motor execution, where the involvement of secondary‐motor regions indicates a motor‐format coding. This interpretation is corroborated by the results of the cross‐regression decoding analysis. In particular, we trained SVRs on the neuronal activation pattern during motor execution and tested for a generalization of these codes in the preceding WM delay. This analysis demonstrates that similar brain activity patterns in the l‐IPS and l‐PMd code an anticipated action, as during the actual execution. These results align with a two‐stage framework of action planning (Boettcher et al. 2021), according to which, after action selection, information about the action outcome is transformed into a motor code during a motor planning stage. The vmPFC likely contributes to action selection (Soon et al. 2008), while the l‐IPS transforms the intended outcome into a motor code (Gallivan and Culham 2015; Wong and Haith 2017; Errante et al. 2021; Klautke et al. 2023). This motor code could then be stored in the PMd and SMA, where specific motor parameters, such as the to‐be used muscle fibres (Mizuguchi et al. 2011, 2013; Mizuguchi, Nakata, and Kanosue 2014), are selected and maintained in preparation for grip‐force execution.

In the following, we will discuss our findings in the temporal sequence from the cue period to the end of the delay period, that is, until motor execution.

### 4.1 The Cue Period

During the cue period, no above‐chance decoding accuracies were found. Only a small cluster within the vmPFC was revealed when inspecting the data at *p* < 0.001 uncorrected, which, however, then becomes significant during the ED period (see Section 4). This finding is consistent with the dynamics of the BOLD response, which is expected to peak at about 3–5 s after event onset, that is, retro‐cue presentation (Martindale et al. 2003; Yeşilyurt, Uğurbil, and Uludağ 2008; Hirano, Stefanovic, and Silva 2011; Hillman 2014). Having found no visual regions suggests that parametric decoding analyses were not influenced by the sensory features of the visual cue, that is, the grip‐force cue, which were experimentally rendered orthogonal to the grip‐force intensities. This finding supports the specificity of the main decoding analysis.

The specificity of our SVR decoding analyses is further corroborated by the results of the control analysis, in which decoding of non‐maintained grip‐force intensities revealed no above‐chance decoding at an uncorrected threshold of *p* < 0.001. Finally, label‐permutation testing demonstrates that prediction accuracies of the main analyses are based on the specific parametric coding of grip‐force intensities. In particular, the computation of parametric contrasts on prediction accuracy maps, which resulted from label permutation testing, strongly corroborates the results of the main analysis, since it shows very similar clusters to the main analysis across the time periods (at *p* < 0.05 FWE corrected). Taken together, these control analyses and null findings indicate a high specificity and sensitivity of the reported findings.

### 4.2 The ED Period: Action Selection in the Ventromedial Prefrontal Cortex

During the ED period, our main decoding analysis revealed a cluster within the vmPFC; specifically, in Brodmann area 10. This finding is consistent with previous fMRI MVPA studies that implicated the vmPFC in prospective memory tasks (Haynes et al. 2007, 2015; Soon et al. 2008, 2013; Gilbert 2011; Momennejad and Haynes 2012). In these studies, participants were instructed to memorize actions that had to be performed upon the presentation of a relevant cue or event (Haynes et al. 2015). Notably, these time‐resolved MVPA studies showed that the contribution of the vmPFC to action‐selection is limited to an initial processing phase right after cue presentation (Soon et al. 2008; Soon et al. 2013).

Alternatively, the vmPFC may have contributed to the maintenance of either a motor intention, that is, the action‐selection stage that precedes movement planning (Ruiz et al. 2024); also referred to as motor decision stage (Soon et al. 2008; Tecilla et al. 2022) or a prospective intention, that is, a mental operation regarding the motor details of an intended action (Soon et al. 2013). The encoding of prospective intentions is typically tested in prospective memory tasks and operationalized as an abstract, future‐oriented memory process involved in the representation and selection of non‐motor actions (Soon et al. 2013). In contrast, the coding of a motor intention underlies a motor‐decision process focused on representing an action outcome and initiating motor planning (Soon et al. 2008). While both cognitive processes could explain above‐chance decoding in the vmPFC (Soon et al. 2008, 2013), the vmPFC's involvement in coding prospective intentions is unlikely due to a key difference between our delayed grip‐force task and traditional prospective memory tasks. Specifically, prospective memory tasks require maintaining intentions while simultaneously performing unrelated secondary tasks during a delay period (Burgess, Scott, and Frith 2003; Gilbert 2011), an absent feature in our task. This distinction complicates direct comparisons with our findings. Conversely, the similarity of our task with those of previous MVPA studies (Soon et al. 2008; Ruiz et al. 2024) renders above‐chance decoding in the vmPFC more likely explained by a contribution to selecting a motor action (Soon et al. 2008; Chatham et al. 2014, 2015; Tecilla et al. 2022) or the (prospective) motor intentions (Hogeveen et al. 2022; Ruiz et al. 2024).

The time course of the prediction accuracy in the vmPFC, which immediately increases and peaks during the ED period (similarly to an evoked BOLD response), further suggests that the information processing underlying action‐selection begins during the cue period and extends into the ED period. This finding aligns with our cross‐decoding analysis, which indicates stable brain activity patterns in the vmPFC throughout these periods. Consequently, this activity can be viewed as the initial stage of action planning, reflecting the selection of an intended action that is subsequently transformed into a more detailed motor plan (Boettcher et al. 2021).

### 4.3 The LD Period: Motor Planning and Grip‐Force Anticipation

Analysis of the LD period revealed distinct regions compared to the ED period, namely: the l‐PMd, l‐IPS, and SMA; that is, regions that are well‐known for their role in motor planning.

The most pronounced cluster was within the l‐PMd. This finding is in agreement with the results of previous fMRI and TMS studies, which reported the involvement of the PMd in the anticipatory scaling of grip‐force (Cole and Rotella 2002; Chouinard, Leonard, and Paus 2005; Nowak et al. 2009; Van Nuenen et al. 2012). In an fMRI TMS study, van Nuenen et al. (2012) performed univariate analysis, which revealed BOLD signal covariation with the cued grip‐force intensities during a delay period. The additional application of low‐frequency repetitive TMS (rTMS) over the l‐PMd corroborated its involvement in grip‐force intensity anticipation (Chouinard, Leonard, and Paus 2005; Nowak et al. 2009; Van Nuenen et al. 2012). Specifically, rTMS modulated the impact of an incorrect predictive cue on subsequent grip‐force execution only when rTMS was applied over the PMd, but not over M1 (Ward et al. 2010; Van Nuenen et al. 2012). This demonstrated that the covariation of the PMd activity with the cued grip‐force intensity is essential for accurately anticipating a to‐be‐executed grip force. A similar contribution of the PMd is suggested by another fMRI study (Mizuguchi, Nakata, and Kanosue 2014), which showed brain activity covariation with three imagined grip‐force intensities. Our fMRI MVPA study extended these works by employing a multivariate rather than a univariate approach, and by showing parametric modulation of the l‐PMd before motor execution. By applying an SVR approach, we directly tested for multivariate codes that map onto a continuous variable, namely, the four anticipated grip‐force intensities. Using label‐permutation tests, we demonstrated the specificity of the activation patterns within PMd for the particular retained intensity. This code within the PMd is plausible when considering its functional contribution to the representation of anticipating the outcome of a motor movement (Gallivan et al. 2011a; Gallivan et al. 2011b; Gallivan et al. 2013; Ariani, Oosterhof, and Lingnau 2018; Errante et al. 2021; Ariani, Pruszynski, and Diedrichsen 2022), an effector‐independent, action‐specific motor plan (Gallivan et al. 2011a; Gallivan et al. 2011b; Gallivan et al. 2013; Gallivan and Culham 2015; Ariani, Wurm, and Lingnau 2015; Ariani, Oosterhof, and Lingnau 2018), and the maintenance of motor movement plans (Hoshi and Tanji 2006; Van Nuenen et al. 2012; Langner et al. 2014).

Beneath the l‐PMd, activation patterns in the l‐IPS were predictive of grip‐force intensities during the LD period. Previous fMRI MVPA studies have consistently shown that the IPS is part of a parieto‐frontal network involved in planning grasping actions, even in the absence of fingers pre‐shaping (Johnson‐Frey, Newman‐Norlund, and Grafton 2005; Tunik, Frey, and Grafton 2005; Davare et al. 2009; Gallivan et al. 2011a; Gallivan et al. 2011b; Gallivan et al. 2013; Ariani, Oosterhof, and Lingnau 2018; Ruiz et al. 2024; Gallivan and Wood 2009; Cavina‐Pratesi et al. 2010). These studies have found anticipatory brain activity in the IPS and decoded movement properties, such as the grip‐type during the delay period (Gallivan et al. 2011a; Gallivan et al. 2013; Ariani, Oosterhof, and Lingnau 2018; Gallivan et al. 2011b; Ariani, Wurm, and Lingnau 2015). Interestingly, time‐resolved decoding analyses have also revealed faster increases of prediction accuracy in the IPS, as compared to effector‐specific brain regions (e.g., the SMA) (Gallivan et al. 2013; Ariani, Oosterhof, and Lingnau 2018). These findings implicate the IPS into the earlier stages of motor planning, which might even precede motor‐preparation (Churchland et al. 2010; Shenoy, Sahani, and Churchland 2013; Gallivan and Culham 2015; Wong and Haith 2017; Ariani, Oosterhof, and Lingnau 2018; Vyas et al. 2020; Ariani, Pruszynski, and Diedrichsen 2022; Ruiz et al. 2024). In particular, the posterior IPS (pIPS) has been shown to represent motor plans in an effector‐independent way and to transform intended motor‐action outcomes into specific movement plans (Gallivan et al. 2011a; Gallivan et al. 2011b; Gallivan et al. 2013; Barany et al. 2014; Gallivan and Culham 2015). This transformation is likely guided by top‐down attentional mechanisms, critical for activating and maintaining motor codes in the PMd and selecting to‐be executed movements (Schmidt and Blankenburg 2019; Calton, Dickinson, and Snyder 2002; Beurze et al. 2009; Szczepanski et al. 2010; Chapman et al. 2011; Gallivan et al. 2011a; Gallivan et al. 2011b). According to this framework, the pIPS acts as a hub for information processing, where it may transform abstract action outcomes (i.e., the intended action selected in the vmPFC) into detailed movement plans (maintained in the PMd) (Gallivan and Culham 2015). These findings align with our results, suggesting that the l‐IPS may specify intended grip‐force intensity in motor plans and support the anticipation and maintenance of motor parameters in the l‐PMd by abstractly coding and transforming the intended grip‐force intensity into a movement plan (Gallivan et al. 2011a; Gallivan et al. 2011b; Gallivan et al. 2013; Gallivan and Culham 2015).

The functional contribution of the IPS (and PMd) in the maintenance of grip‐force intensities is further supported by neurophysiological studies focused on motor‐WM (Passingham 1988, 1989; Hoshi and Tanji 2000; Hoshi and Tanji 2006; Passingham et al. 2007; Nakayama et al. 2008; Langner et al. 2014; Formica et al. 2021). In particular, it has been shown that anatomical and functional connections between the IPS and the PMd facilitate the maintenance of motor information during a delay period (Matelli et al. 1986; Johnson‐Frey, Newman‐Norlund, and Grafton 2005; Wise, Di Pellegrino, and Boussaoud 1996; Wise et al. 1997; Toni, Rushworth, and Passingham 2001; Hoshi and Tanji 2004a; Hoshi and Tanji 2004b; Hoshi and Tanji 2006; Pardo‐Vazquez et al. 2011). While the IPS contributes to the early specification of the to‐be executed motor plan (Heed et al. 2016; Klautke et al. 2023; Ruiz et al. 2024), the PMd contributes to the maintenance of effector‐specific movement parameters (Passingham 1988, 1989; Kurata and Hoffman 1994; Riehle and Requin 1989; Requin and Riehle 1995; Hoshi and Tanji 2006; Heuer et al. 2020).

The network of brain regions from which grip‐force intensity can be decoded during the delay period showed a third peak in the SMA. The involvement of the SMA in motor preparation has been extensively demonstrated (Marsden et al. 1996; Passingham 1996; Picard and Strick 1996; Rizzolatti et al. 1996; Petit et al. 1998; Lee, Chang, and Roh 1999; Sreenivasan and D'Esposito 2019; Bonicalzi and Haggard 2019), including the preparation of grip‐force intensity (Van Nuenen et al. 2012). It appears plausible that the l‐PMd, in interaction with SMA, is involved in generating ready‐to‐use movement codes (Mizuguchi et al. 2011, 2013; Van Ede and Nobre 2023). This is supported by the presence of catch trials requiring rapid and precise grip‐force execution, which presuppose the anticipation and maintenance of the intended grip‐force in a ready‐to‐use format (Boettcher et al. 2021).

In conclusion, the involvement of pre‐ and supplementary motor cortices during the LD period reinforces the view that information is stored in a format—or by the same neuronal populations—as motor movements themselves (Mizuguchi et al. 2011, 2013). This hypothesis is corroborated by our cross‐regression decoding analysis, which revealed overlapping clusters in the l‐IPS and l‐PMd during both the LD period and motor execution. Since executing a motor movement requires transforming action‐specific information into a motor code, and a similar code is found during the LD period, it is likely that information about motor movements was coded well before execution. Interestingly, while generalization to the cue period only showed above‐chance decoding in the vmPFC, the LD period also showed the l‐PMd and l‐IPS. This difference is likely due to privileged anatomical connections among prefrontal, premotor, and parietal brain regions (Ridderinkhof et al. 2004; Huang et al. 2019; Löffler, Haggard, and Bode 2020; Ruiz et al. 2024), with the l‐PMd serving as a central hub in motor planning and movement preparation (Ruiz et al. 2024). This suggests that, once motor decisions are made in the vmPFC, intended grip‐force intensities are transformed in the l‐IPS and maintained in motor codes of the l‐PMd, which is where movement planning occurs. The late increase in prediction accuracy observed in the SMA (at 9 s from cue presentation), combined with below‐chance cross‐regression decoding accuracy in this area may suggest the SMA's contribution to initiating motor movements (Gallivan and Culham 2015; Wong and Haith 2017; Ariani, Oosterhof, and Lingnau 2018; Vyas et al. 2020).

### 4.4 Motor Execution

During the motor execution period, our parametric decoding showed above‐chance decoding in several brain regions: bilateral primary motor cortices (M1), the l‐IPS, l‐PMd, SMA, and S1 (at *p* < 0.05 FWE‐corrected). These results are consistent with previous fMRI studies, showing covariation of M1, SMA, and l‐S1 activity with the executed grip‐force intensity (Dettmers et al. 1995; Thickbroom et al. 1998; Dai et al. 2001; Ehrsson, Fagergren, and Forssberg 2001; Cramer et al. 2002; Andrushko et al. 2021). The involvement of the M1 and S1 during motor execution, alongside brain‐regions found during the LD period (i.e., the l‐IPS, l‐PMd, and SMA), is expected. The M1 is well‐known for its role in generating motor output (Dechent, Merboldt, and Frahm 2004), while S1 plays a crucial role in processing sensory feedback essential for motor control (Wolpert and Flanagan 2001).

Interestingly, while movement‐specific information can be decoded from secondary motor regions during both planning and motor execution, above‐chance decoding in the M1 is typically limited to the execution period (Ruiz et al. 2024). This finding aligns with the results of our main and cross‐regression decoding analyses, which did not reveal significant involvement of M1 before motor execution (at *p* < 0.05 FWE‐corrected). However, this is only partially consistent with previous fMRI cross‐decoding findings (Ariani, Oosterhof, and Lingnau 2018). Specifically, Ariani, Oosterhof, and Lingnau (2018) observed above‐chance decoding in both PMd and M1 by cross‐decoding between delayed and non‐delayed reach‐to‐grasp tasks. Their delayed condition involved planning and controlling finger kinematics during hand pre‐shaping and grasping. Since many neurons in M1 are selective for hand kinematics, this may explain why M1 was engaged in both planning and execution in their study. In contrast, our isometric grip‐force task, which does not require finger kinematics planning, could explain the absence of significant M1 involvement until actual execution. The differences between their findings and ours, particularly the lack of above‐chance decoding in M1 during motor planning, may be attributed to the distinct task demands in each study (Monaco et al. 2020).

In conclusion, results from our parametric analyses corroborate the hypothesis that grip‐force anticipation primarily involves the PMd, and not M1 or SMA (Van Nuenen et al. 2012), indicating a separation of planning and execution functions (Ruiz et al. 2024). This differentiation further supports the notion that M1 is more engaged in generating the actual motor output rather than maintaining anticipatory codes (Ruiz et al. 2024).

### 4.5 Task‐Demand‐Dependent WM Codes: Prospective Versus Retrospective Working Memory

WM representations adapt to the task demands, which employ different neural codes depending on whether the task requires the storage of sensory information (for future comparison with test stimuli) or the transformation of action‐specific information into motor movements (Lee et al. 2013; Myers, Stokes, and Nobre 2017; Christophel et al. 2017; Nobre and Stokes 2019). While sensory‐WM tasks are used to test the retention of sensory information (Schmidt, Wu, and Blankenburg 2017; Uluç et al. 2018; Wu et al. 2018; Uluç et al. 2020), the present study provides critical insights into how WM content is translated into a motor output. In particular, our parametric analyses reveal that information about grip‐force intensities is encoded and maintained in distinct brain activity patterns and representational formats across different time‐periods of the task. Notably, the presence of similar motor codes during the LD and motor execution periods supports our hypothesis that intended grip‐force intensities were transformed and maintained in a ready‐to‐use motor format. The presence of WM codes in secondary motor regions corroborates the prospective and action‐oriented nature of the undergone task performance.

The action‐oriented, prospective nature of our delayed grip‐force task allow an interesting comparison with previous research on parametric WM representations, which typically investigate retrospective WM (see Burgess et al. 2011; Mackey and Curtis 2017; Christophel et al. 2017; Van Ede and Nobre 2023 for further discussion of retrospective and prospective WM functions). Building on the seminal work of Romo et al. (1999) in non‐human primates, a series of human WM studies have explored different types of parametric WM content. This work has confirmed the relevance of the right inferior frontal gyrus (r‐IFG) for the retention of parametric vibratory (Spitzer, Wacker, and Blankenburg 2010; Spitzer et al. 2014a; Schmidt, Wu, and Blankenburg 2017), auditory (Spitzer and Blankenburg 2012; Spitzer et al. 2014b; Uluç et al. 2018) as well as visual flicker frequency (Spitzer and Blankenburg 2012; Wu et al. 2018). The functional role of the r‐IFG in parametric WM is typically interpreted as a reflection of abstract codes of supramodal quantity (Walsh 2003; Cohen Kadosh and Walsh 2009). Interestingly, we did not find significant above‐chance decoding in the IFG (at *p* < 0.05 FWE), even when using more liberal thresholds (at *p* < 0.001 uncorrected). This is a noteworthy difference when aiming to distinguish between retrospective and prospective WM functions. Indeed, it suggests that parametric content representations in the r‐IFG are characteristic of retrospective WM, thereby acting as an information buffer for previously perceived data. This information is then maintained in a format suited for abstract processing rather than being directly translated into a motor code. Vice‐versa, the reported involvement of the vmPFC in a time‐period that precedes transformation into a motor code (and the above‐chance decoding in secondary motor regions) may suggest that the vmPFC contributes to the maintenance of parametric, action‐specific information for direct translation into motor plans. After translation, this code may be maintained in the PMd. Therefore, our study complements existing literature by revealing that parametric WM codes can retain action‐specific information before, and after translation into a motor‐code.

## 5 Conclusion

In this study, we used MVPA to identify a network of brain regions that encodes parametric WM for the selection, planning and anticipation of to‐be executed grip‐force intensities. We showed that, after action selection, information on the intended grip‐force is transformed into a motor code during a motor planning phase. Specifically, the vmPFC is likely involved in action selection (Soon et al. 2008), while the l‐IPS contributed to converting this information into a motor code (Gallivan and Culham 2015; Wong and Haith 2017; Errante et al. 2021; Klautke et al. 2023). This motor code is then maintained in the PMd, for the anticipation of grip‐force execution in interaction with the SMA. Consistently, cross‐regression decoding shows similar activation patterns in the l‐PMd and l‐IPS during both the LD period and motor execution, which indicates that action‐specific information is retained in a format akin to the motor movement itself (i.e., a motor format; Butterfill and Sinigaglia 2014; Mylopoulos and Pacherie 2019).

Our findings align with the view that different types of WM codes are used depending on the task demands (Christophel et al. 2017). While much of WM research is focused on sensory‐type information in retrospective WM (Schmidt, Wu, and Blankenburg 2017; Uluç et al. 2018; Wu et al. 2018; Uluç et al. 2020), the study at hand is an important step to distinguish and characterize how WM content is translated into prospective motor output.

## Ethics Statement

Participants gave their written consent before proceeding with the experiment. The experimental design and materials were approved by the ethics committee at Freie Universität Berlin (003/2021, Berlin, Germany).

## Conflicts of Interest

The authors declare no conflicts of interest.

## Supporting information


**DATA S1** Supporting Information.

## Data Availability

The data that support the findings of this study are available on request from the corresponding author. The data are not publicly available due to privacy or ethical restrictions.

## References

[hbm70154-bib-0002] Ali, Y. , V. Montani , and P. Cesari . 2023. “The Touch in Action: Exploring Sensorimotor Interactions With Motor Imagery.” Cerebral Cortex 33, no. 13: 8382–8390. 10.1093/cercor/bhad123. 37032623

[hbm70154-bib-0004] Andersen, R. A. , and H. Cui . 2009. “Intention, Action Planning, and Decision Making in Parietal‐Frontal Circuits.” Neuron 63, no. 5: 568–583. 10.1016/j.neuron.2009.08.028. 19755101

[hbm70154-bib-0005] Andrushko, J. W. , L. A. Gould , D. W. Renshaw , et al. 2021. “High Force Unimanual Handgrip Contractions Increase Ipsilateral Sensorimotor Activation and Functional Connectivity.” Neuroscience 452: 111–125. 10.1016/j.neuroscience.2020.10.031. 33197497

[hbm70154-bib-0006] Ariani, G. , N. Kordjazi , J. A. Pruszynski , and J. Diedrichsen . 2021. “The Planning Horizon for Movement Sequences.” Eneuro 8, no. 2: ENEURO.0085‐21.2021. 10.1523/ENEURO.0085-21.2021. PMC8174040 33753410

[hbm70154-bib-0007] Ariani, G. , N. N. Oosterhof , and A. Lingnau . 2018. “Time‐Resolved Decoding of Planned Delayed and Immediate Prehension Movements.” Cortex 99: 330–345. 10.1016/j.cortex.2017.12.007. 29334647

[hbm70154-bib-0008] Ariani, G. , J. A. Pruszynski , and J. Diedrichsen . 2022. “Motor Planning Brings Human Primary Somatosensory Cortex Into Action‐Specific Preparatory States.” eLife 11: e69517. 10.7554/eLife.69517. 35018886 PMC8786310

[hbm70154-bib-0009] Ariani, G. , M. Shahbazi , and J. Diedrichsen . 2024. “Cortical Areas for Planning Sequences Before and During Movement.” Journal of Neuroscience 45, no. 3: e1300242024. 10.1523/JNEUROSCI.1300-24.2024. PMC11735648 39542728

[hbm70154-bib-0010] Ariani, G. , M. F. Wurm , and A. Lingnau . 2015. “Decoding Internally and Externally Driven Movement Plans.” Journal of Neuroscience 35, no. 42: 14160–14171. 10.1523/JNEUROSCI.0596-15.2015. 26490857 PMC6605426

[hbm70154-bib-0013] Barany, D. A. , V. Della‐Maggiore , S. Viswanathan , M. Cieslak , and S. T. Grafton . 2014. “Feature Interactions Enable Decoding of Sensorimotor Transformations for Goal‐Directed Movement.” Journal of Neuroscience 34, no. 20: 6860–6873. 10.1523/JNEUROSCI.5173-13.2014. 24828640 PMC4099499

[hbm70154-bib-0014] Beurze, S. M. , F. P. De Lange , I. Toni , and W. P. Medendorp . 2009. “Spatial and Effector Processing in the Human Parietofrontal Network for Reaches and Saccades.” Journal of Neurophysiology 101, no. 6: 3053–3062. 10.1152/jn.91194.2008. 19321636

[hbm70154-bib-0015] Bode, S. , and J.‐D. Haynes . 2009. “Decoding Sequential Stages of Task Preparation in the Human Brain.” NeuroImage 45, no. 2: 606–613. 10.1016/j.neuroimage.2008.11.031. 19111624

[hbm70154-bib-0016] Bode, S. , E. Schubert , H. Hogendoorn , and D. Feuerriegel . 2022. “Decoding Continuous Variables From Event‐Related Potential (ERP) Data With Linear Support Vector Regression Using the Decision Decoding Toolbox (DDTBOX).” Frontiers in Neuroscience 16: 989589. 10.3389/fnins.2022.989589. 36408410 PMC9669708

[hbm70154-bib-0017] Boettcher, S. E. P. , D. Gresch , A. C. Nobre , and F. Van Ede . 2021. “Output Planning at the Input Stage in Visual Working Memory.” Science Advances 7, no. 13: eabe8212. 10.1126/sciadv.abe8212. 33762341 PMC7990334

[hbm70154-bib-0018] Bonicalzi, S. , and P. Haggard . 2019. “From Freedom From to Freedom to: New Perspectives on Intentional Action.” Frontiers in Psychology 10: 1193. 10.3389/fpsyg.2019.01193. 31191396 PMC6546819

[hbm70154-bib-0019] Brainard, D. H. 1997. “The Psychophysics Toolbox.” Spatial Vision 10, no. 4: 433–436. 10.1163/156856897X00357. 9176952

[hbm70154-bib-0020] Brody, C. D. 2003. “Timing and Neural Encoding of Somatosensory Parametric Working Memory in Macaque Prefrontal Cortex.” Cerebral Cortex 13, no. 11: 1196–1207. 10.1093/cercor/bhg100. 14576211

[hbm70154-bib-0021] Burgess, P. W. , G. Gonen‐Yaacovi , and E. Volle . 2011. “Functional Neuroimaging Studies of Prospective Memory: What Have We Learnt So Far?” Neuropsychologia 49, no. 8: 2246–2257. 10.1016/j.neuropsychologia.2011.02.014. 21329712

[hbm70154-bib-0022] Burgess, P. W. , S. K. Scott , and C. D. Frith . 2003. “The Role of the Rostral Frontal Cortex (Area 10) in Prospective Memory: A Lateral Versus Medial Dissociation.” Neuropsychologia 41, no. 8: 906–918. 10.1016/S0028-3932(02)00327-5. 12667527

[hbm70154-bib-0023] Butterfill, S. A. , and C. Sinigaglia . 2014. “Intention and Motor Representation in Purposive Action.” Philosophy and Phenomenological Research 88, no. 1: 119–145. 10.1111/j.1933-1592.2012.00604.x.

[hbm70154-bib-0024] Calton, J. L. , A. R. Dickinson , and L. H. Snyder . 2002. “Non‐Spatial, Motor‐Specific Activation in Posterior Parietal Cortex.” Nature Neuroscience 5, no. 6: 580–588. 10.1038/nn0602-862. 12021766

[hbm70154-bib-0025] Cattaneo, L. , F. Caruana , A. Jezzini , and G. Rizzolatti . 2009. “Representation of Goal and Movements Without Overt Motor Behavior in the Human Motor Cortex: A Transcranial Magnetic Stimulation Study.” Journal of Neuroscience 29, no. 36: 11134–11138. 10.1523/JNEUROSCI.2605-09.2009. 19741119 PMC6665924

[hbm70154-bib-0027] Cavina‐Pratesi, C. , S. Monaco , P. Fattori , et al. 2010. “Functional Magnetic Resonance Imaging Reveals the Neural Substrates of Arm Transport and Grip Formation in Reach‐to‐Grasp Actions in Humans.” Journal of Neuroscience 30, no. 31: 10306–10323. 10.1523/JNEUROSCI.2023-10.2010. 20685975 PMC6634677

[hbm70154-bib-0029] Chapman, C. S. , J. P. Gallivan , J. C. Culham , and M. A. Goodale . 2011. “Mental Blocks: fMRI Reveals Top‐Down Modulation of Early Visual Cortex When Obstacles Interfere With Grasp Planning.” Neuropsychologia 49, no. 7: 1703–1717. 10.1016/j.neuropsychologia.2011.02.048. 21376065

[hbm70154-bib-0030] Chatham, C. H. , and D. Badre . 2015. “Multiple Gates on Working Memory.” Current Opinion in Behavioral Sciences 1: 23–31. 10.1016/j.cobeha.2014.08.001. 26719851 PMC4692183

[hbm70154-bib-0031] Chatham, C. H. , M. J. Frank , and D. Badre . 2014. “Corticostriatal Output Gating During Selection From Working Memory.” Neuron 81, no. 4: 930–942. 10.1016/j.neuron.2014.01.002. 24559680 PMC3955887

[hbm70154-bib-0032] Chouinard, P. A. , G. Leonard , and T. Paus . 2005. “Role of the Primary Motor and Dorsal Premotor Cortices in the Anticipation of Forces During Object Lifting.” Journal of Neuroscience 25, no. 9: 2277–2284. 10.1523/JNEUROSCI.4649-04.2005. 15745953 PMC6726079

[hbm70154-bib-0033] Christophel, T. B. , R. M. Cichy , M. N. Hebart , and J.‐D. Haynes . 2015. “Parietal and Early Visual Cortices Encode Working Memory Content Across Mental Transformations.” NeuroImage 106: 198–206. 10.1016/j.neuroimage.2014.11.018. 25463456

[hbm70154-bib-0034] Christophel, T. B. , M. N. Hebart , and J.‐D. Haynes . 2012. “Decoding the Contents of Visual Short‐Term Memory From Human Visual and Parietal Cortex.” Journal of Neuroscience 32, no. 38: 12983–12989. 10.1523/JNEUROSCI.0184-12.2012. 22993415 PMC6621473

[hbm70154-bib-0035] Christophel, T. B. , P. C. Klink , B. Spitzer , P. R. Roelfsema , and J.‐D. Haynes . 2017. “The Distributed Nature of Working Memory.” Trends in Cognitive Sciences 21, no. 2: 111–124. 10.1016/j.tics.2016.12.007. 28063661

[hbm70154-bib-0036] Churchland, M. M. , J. P. Cunningham , M. T. Kaufman , S. I. Ryu , and K. V. Shenoy . 2010. “Cortical Preparatory Activity: Representation of Movement or First Cog in a Dynamical Machine?” Neuron 68, no. 3: 387–400. 10.1016/j.neuron.2010.09.015. 21040842 PMC2991102

[hbm70154-bib-0037] Cisek, P. , and J. F. Kalaska . 2004. “Neural Correlates of Mental Rehearsal in Dorsal Premotor Cortex.” Nature 431, no. 7011: 993–996. 10.1038/nature03005. 15496925

[hbm70154-bib-0038] Cisek, P. , and J. F. Kalaska . 2005. “Neural Correlates of Reaching Decisions in Dorsal Premotor Cortex: Specification of Multiple Direction Choices and Final Selection of Action.” Neuron 45, no. 5: 801–814. 10.1016/j.neuron.2005.01.027. 15748854

[hbm70154-bib-0039] Cisek, P. , and J. F. Kalaska . 2010. “Neural Mechanisms for Interacting With a World Full of Action Choices.” Annual Review of Neuroscience 33, no. 1: 269–298. 10.1146/annurev.neuro.051508.135409. 20345247

[hbm70154-bib-0040] Cohen Kadosh, R. , and V. Walsh . 2009. “Numerical Representation in the Parietal Lobes: Abstract or Not Abstract?” Behavioral and Brain Sciences 32, no. 3–4: 313–328. 10.1017/S0140525X09990938. 19712504

[hbm70154-bib-0043] Cole, K. J. , and D. L. Rotella . 2002. “Old Age Impairs the Use of Arbitrary Visual Cues for Predictive Control of Fingertip Forces During Grasp.” Experimental Brain Research 143, no. 1: 35–41. 10.1007/s00221-001-0965-9. 11907688

[hbm70154-bib-0045] Cramer, S. C. , R. M. Weisskoff , J. D. Schaechter , et al. 2002. “Motor Cortex Activation Is Related to Force of Squeezing.” Human Brain Mapping 16, no. 4: 197–205. 10.1002/hbm.10040. 12112762 PMC6871791

[hbm70154-bib-0047] Cui, H. , and R. A. Andersen . 2007. “Posterior Parietal Cortex Encodes Autonomously Selected Motor Plans.” Neuron 56, no. 3: 552–559. 10.1016/j.neuron.2007.09.031. 17988637 PMC2651089

[hbm70154-bib-0048] Cui, H. , and R. A. Andersen . 2011. “Different Representations of Potential and Selected Motor Plans by Distinct Parietal Areas.” Journal of Neuroscience 31, no. 49: 18130–18136. 10.1523/JNEUROSCI.6247-10.2011. 22159124 PMC3327481

[hbm70154-bib-0049] Dai, T. , J. Liu , V. Sahgal , R. Brown , and G. Yue . 2001. “Relationship Between Muscle Output and Functional MRI‐Measured Brain Activation.” Experimental Brain Research 140, no. 3: 290–300. 10.1007/s002210100815. 11681304

[hbm70154-bib-0050] Davare, M. , K. Montague , E. Olivier , J. C. Rothwell , and R. N. Lemon . 2009. “Ventral Premotor to Primary Motor Cortical Interactions During Object‐Driven Grasp in Humans.” Cortex 45, no. 9: 1050–1057. 10.1016/j.cortex.2009.02.011. 19345344 PMC2730595

[hbm70154-bib-0052] Dechent, P. , K.‐D. Merboldt , and J. Frahm . 2004. “Is the Human Primary Motor Cortex Involved in Motor Imagery?” Cognitive Brain Research 19, no. 2: 138–144. 10.1016/j.cogbrainres.2003.11.012. 15019710

[hbm70154-bib-0054] Dettmers, C. , G. R. Fink , R. N. Lemon , et al. 1995. “Relation Between Cerebral Activity and Force in the Motor Areas of the Human Brain.” Journal of Neurophysiology 74, no. 2: 802–815. 10.1152/jn.1995.74.2.802. 7472384

[hbm70154-bib-0057] Ehrsson, H. H. , A. Fagergren , and H. Forssberg . 2001. “Differential Fronto‐Parietal Activation Depending on Force Used in a Precision Grip Task: An fMRI Study.” Journal of Neurophysiology 85, no. 6: 2613–2623. 10.1152/jn.2001.85.6.2613. 11387405

[hbm70154-bib-0059] Errante, A. , S. Ziccarelli , G. P. Mingolla , and L. Fogassi . 2021. “Decoding Grip Type and Action Goal During the Observation of Reaching‐Grasping Actions: A Multivariate fMRI Study.” NeuroImage 243: 118511. 10.1016/j.neuroimage.2021.118511. 34450263

[hbm70154-bib-0227] Fechner, G. T. 1948. “Elements of Psychophysics, 1860.” In Readings in the History of Psychology: 206–213. 10.1037/11304-026.

[hbm70154-bib-0062] Formica, S. , C. González‐García , M. Senoussi , and M. Brass . 2021. “Neural Oscillations Track the Maintenance and Proceduralization of Novel Instructions.” NeuroImage 232: 117870. 10.1016/j.neuroimage.2021.117870. 33607280

[hbm70154-bib-0065] Gallivan, J. P. , and J. C. Culham . 2015. “Neural Coding Within Human Brain Areas Involved in Actions.” Current Opinion in Neurobiology 33: 141–149. 10.1016/j.conb.2015.03.012. 25876179

[hbm70154-bib-0232] Gallivan, J. P. , I. S. Johnsrude , and J. R. Flanagan . 2015. “Planning Ahead: Object‐Directed Sequential Actions Decoded from Human Frontoparietal and Occipitotemporal Networks.” Cerebral Cortex 26: 708–730 bhu302. 10.1093/cercor/bhu302. 25576538 PMC4712801

[hbm70154-bib-0067] Gallivan, J. P. , D. A. McLean , J. R. Flanagan , and J. C. Culham . 2013. “Where One Hand Meets the Other: Limb‐Specific and Action‐Dependent Movement Plans Decoded From Preparatory Signals in Single Human Frontoparietal Brain Areas.” Journal of Neuroscience 33, no. 5: 1991–2008. 10.1523/JNEUROSCI.0541-12.2013. 23365237 PMC6619126

[hbm70154-bib-0068] Gallivan, J. P. , D. A. McLean , F. W. Smith , and J. C. Culham . 2011a. “Decoding Effector‐Dependent and Effector‐Independent Movement Intentions From Human Parieto‐Frontal Brain Activity.” Journal of Neuroscience 31, no. 47: 17149–17168. 10.1523/JNEUROSCI.1058-11.2011. 22114283 PMC6623835

[hbm70154-bib-0069] Gallivan, J. P. , D. A. McLean , K. F. Valyear , C. E. Pettypiece , and J. C. Culham . 2011b. “Decoding Action Intentions From Preparatory Brain Activity in Human Parieto‐Frontal Networks.” Journal of Neuroscience 31, no. 26: 9599–9610. 10.1523/JNEUROSCI.0080-11.2011. 21715625 PMC6623162

[hbm70154-bib-0070] Gallivan, J. P. , and D. K. Wood . 2009. “Simultaneous Encoding of Potential Grasping Movements in Macaque Anterior Intraparietal Area.” Journal of Neuroscience 29, no. 39: 12031–12032. 10.1523/JNEUROSCI.3245-09.2009. 19793960 PMC6666131

[hbm70154-bib-0072] Gilbert, S. J. 2011. “Decoding the Content of Delayed Intentions.” Journal of Neuroscience 31, no. 8: 2888–2894. 10.1523/JNEUROSCI.5336-10.2011. 21414910 PMC6623766

[hbm70154-bib-0078] Hamilton, A. F. , and S. T. Grafton . 2008. “Action Outcomes Are Represented in Human Inferior Frontoparietal Cortex.” Cerebral Cortex 18, no. 5: 1160–1168. 10.1093/cercor/bhm150. 17728264

[hbm70154-bib-0229] Harrison, S. A. , and F. Tong . 2009. “Decoding Reveals the Contents of Visual Working Memory in Early Visual Areas.” Nature 458, no. 7238: 632–635. 10.1038/nature07832. 19225460 PMC2709809

[hbm70154-bib-0080] Haynes, J.‐D. , and G. Rees . 2006. “Decoding Mental States From Brain Activity in Humans.” Nature Reviews Neuroscience 7, no. 7: 523–534. 10.1038/nrn1931. 16791142

[hbm70154-bib-0081] Haynes, J.‐D. , K. Sakai , G. Rees , S. Gilbert , C. Frith , and R. E. Passingham . 2007. “Reading Hidden Intentions in the Human Brain.” Current Biology 17, no. 4: 323–328. 10.1016/j.cub.2006.11.072. 17291759

[hbm70154-bib-0082] Haynes, J.‐D. , D. Wisniewski , K. Gorgen , I. Momennejad , and C. Reverberi . 2015. “FMRI Decoding of Intentions: Compositionality, Hierarchy and Prospective Memory. The 3rd International Winter Conference on Brain‐Computer Interface, 1–3.” 10.1109/IWW-BCI.2015.7073031.

[hbm70154-bib-0083] Hebart, M. N. , and C. I. Baker . 2018. “Deconstructing Multivariate Decoding for the Study of Brain Function.” NeuroImage 180: 4–18. 10.1016/j.neuroimage.2017.08.005. 28782682 PMC5797513

[hbm70154-bib-0084] Hebart, M. N. , B. B. Bankson , A. Harel , C. I. Baker , and R. M. Cichy . 2018. “The Representational Dynamics of Task and Object Processing in Humans.” eLife 7: e32816. 10.7554/eLife.32816. 29384473 PMC5811210

[hbm70154-bib-0233] Hebart, M. N. , K. Görgen , and J.‐D. Haynes . 2015. “The Decoding Toolbox (TDT): A Versatile Software Package for Multivariate Analyses of Functional Imaging Data.” Frontiers in Neuroinformatics 8. 10.3389/fninf.2014.00088. PMC4285115 25610393

[hbm70154-bib-0087] Heed, T. , F. T. M. Leone , I. Toni , and W. P. Medendorp . 2016. “Functional Versus Effector‐Specific Organization of the Human Posterior Parietal Cortex: Revisited.” Journal of Neurophysiology 116, no. 4: 1885–1899. 10.1152/jn.00312.2014. 27466132 PMC5144691

[hbm70154-bib-0089] Heuer, A. , S. Ohl , and M. Rolfs . 2020. “Memory for Action: A Functional View of Selection in Visual Working Memory.” Visual Cognition 28, no. 5–8: 388–400. 10.1080/13506285.2020.1764156.

[hbm70154-bib-0090] Hillman, E. M. C. 2014. “Coupling Mechanism and Significance of the BOLD Signal: A Status Report.” Annual Review of Neuroscience 37, no. 1: 161–181. 10.1146/annurev-neuro-071013-014111. PMC4147398 25032494

[hbm70154-bib-0091] Hirano, Y. , B. Stefanovic , and A. C. Silva . 2011. “Spatiotemporal Evolution of the Functional Magnetic Resonance Imaging Response to Ultrashort Stimuli.” Journal of Neuroscience 31, no. 4: 1440–1447. 10.1523/JNEUROSCI.3986-10.2011. 21273428 PMC3078723

[hbm70154-bib-0092] Hogeveen, J. , M. Medalla , M. Ainsworth , et al. 2022. “What Does the Frontopolar Cortex Contribute to Goal‐Directed Cognition and Action?” Journal of Neuroscience 42, no. 45: 8508–8513. 10.1523/JNEUROSCI.1143-22.2022. 36351824 PMC9665930

[hbm70154-bib-0096] Hoshi, E. , and J. Tanji . 2000. “Integration of Target and Body‐Part Information in the Premotor Cortex When Planning Action.” Nature 408, no. 6811: 466–470. 10.1038/35044075. 11100727

[hbm70154-bib-0097] Hoshi, E. , and J. Tanji . 2004a. “Area‐Selective Neuronal Activity in the Dorsolateral Prefrontal Cortex for Information Retrieval and Action Planning.” Journal of Neurophysiology 91, no. 6: 2707–2722. 10.1152/jn.00904.2003. 14749313

[hbm70154-bib-0098] Hoshi, E. , and J. Tanji . 2004b. “Differential Roles of Neuronal Activity in the Supplementary and Presupplementary Motor Areas: From Information Retrieval to Motor Planning and Execution.” Journal of Neurophysiology 92, no. 6: 3482–3499. 10.1152/jn.00547.2004. 15269227

[hbm70154-bib-0099] Hoshi, E. , and J. Tanji . 2006. “Differential Involvement of Neurons in the Dorsal and Ventral Premotor Cortex During Processing of Visual Signals for Action Planning.” Journal of Neurophysiology 95, no. 6: 3596–3616. 10.1152/jn.01126.2005. 16495361

[hbm70154-bib-0100] Hoshi, E. , and J. Tanji . 2007. “Distinctions Between Dorsal and Ventral Premotor Areas: Anatomical Connectivity and Functional Properties.” Current Opinion in Neurobiology 17, no. 2: 234–242. 10.1016/j.conb.2007.02.003. 17317152

[hbm70154-bib-0101] Huang, Y. , J. Hullfish , D. De Ridder , and S. Vanneste . 2019. “Meta‐Analysis of Functional Subdivisions Within Human Posteromedial Cortex.” Brain Structure and Function 224, no. 1: 435–452. 10.1007/s00429-018-1781-3. 30367247

[hbm70154-bib-0104] Johnson‐Frey, S. H. , R. Newman‐Norlund , and S. T. Grafton . 2005. “A Distributed Left Hemisphere Network Active During Planning of Everyday Tool Use Skills.” Cerebral Cortex 15, no. 6: 681–695. 10.1093/cercor/bhh169. 15342430 PMC1364509

[hbm70154-bib-0105] Jones, L. A. 1989. “Matching Forces: Constant Errors and Differential Thresholds.” Perception 18, no. 5: 681–687. 10.1068/p180681. 2602094

[hbm70154-bib-0106] Kahnt, T. , J. Heinzle , S. Q. Park , and J.‐D. Haynes . 2011. “Decoding Different Roles for vmPFC and dlPFC in Multi‐Attribute Decision Making.” NeuroImage 56, no. 2: 709–715. 10.1016/j.neuroimage.2010.05.058. 20510371

[hbm70154-bib-0110] Kim, H. E. , G. Avraham , and R. B. Ivry . 2021. “The Psychology of Reaching: Action Selection, Movement Implementation, and Sensorimotor Learning.” Annual Review of Psychology 72, no. 1: 61–95. 10.1146/annurev-psych-010419-051053. PMC8514106 32976728

[hbm70154-bib-0111] King, J.‐R. , and S. Dehaene . 2014. “Characterizing the Dynamics of Mental Representations: The Temporal Generalization Method.” Trends in Cognitive Sciences 18, no. 4: 203–210. 10.1016/j.tics.2014.01.002. 24593982 PMC5635958

[hbm70154-bib-0112] Klautke, J. , C. Foster , W. P. Medendorp , and T. Heed . 2023. “Dynamic Spatial Coding in Parietal Cortex Mediates Tactile‐Motor Transformation.” Nature Communications 14, no. 1: 4532. 10.1038/s41467-023-39959-4. PMC10374589 37500625

[hbm70154-bib-0116] Kurata, K. , and D. S. Hoffman . 1994. “Differential Effects of Muscimol Microinjection Into Dorsal and Ventral Aspects of the Premotor Cortex of Monkeys.” Journal of Neurophysiology 71, no. 3: 1151–1164. 10.1152/jn.1994.71.3.1151. 8201409

[hbm70154-bib-0119] Langner, R. , M. A. Sternkopf , T. S. Kellermann , et al. 2014. “Translating Working Memory Into Action: Behavioral and Neural Evidence for Using Motor Representations in Encoding Visuo‐Spatial Sequences: Motor Working Memory.” Human Brain Mapping 35, no. 7: 3465–3484. 10.1002/hbm.22415. 24222405 PMC6869028

[hbm70154-bib-0120] Lee, K.‐M. , K.‐H. Chang , and J.‐K. Roh . 1999. “Subregions Within the Supplementary Motor Area Activated at Different Stages of Movement Preparation and Execution.” NeuroImage 9, no. 1: 117–123. 10.1006/nimg.1998.0393. 9918733

[hbm70154-bib-0122] Lee, S.‐H. , D. J. Kravitz , and C. I. Baker . 2013. “Goal‐Dependent Dissociation of Visual and Prefrontal Cortices During Working Memory.” Nature Neuroscience 16, no. 8: 997–999. 10.1038/nn.3452. 23817547 PMC3781947

[hbm70154-bib-0123] Leoné, F. T. M. , T. Heed , I. Toni , and W. P. Medendorp . 2014. “Understanding Effector Selectivity in Human Posterior Parietal Cortex by Combining Information Patterns and Activation Measures.” Journal of Neuroscience 34, no. 21: 7102–7112. 10.1523/JNEUROSCI.5242-13.2014. 24849346 PMC6608188

[hbm70154-bib-0127] Löffler, A. , P. Haggard , and S. Bode . 2020. “Decoding Changes of Mind in Voluntary Action—Dynamics of Intentional Choice Representations.” Cerebral Cortex 30, no. 3: 1199–1212. 10.1093/cercor/bhz160. 31504263

[hbm70154-bib-0128] Mackey, W. E. , and C. E. Curtis . 2017. “Distinct Contributions by Frontal and Parietal Cortices Support Working Memory.” Scientific Reports 7, no. 1: 6188. 10.1038/s41598-017-06293-x. 28733684 PMC5522403

[hbm70154-bib-0130] Marsden, C. D. , L. Deecke , H. J. Freund , et al. 1996. “The Functions of the Supplementary Motor Area. Summary of a Workshop.” Advances in Neurology 70: 477–487. 8615229

[hbm70154-bib-0131] Martindale, J. , J. Mayhew , J. Berwick , et al. 2003. “The Hemodynamic Impulse Response to a Single Neural Event.” Journal of Cerebral Blood Flow & Metabolism 23, no. 5: 546–555. 10.1097/01.WCB.0000058871.46954.2B. 12771569

[hbm70154-bib-0132] Matelli, M. , R. Camarda , M. Glickstein , and G. Rizzolatti . 1986. “Afferent and Efferent Projections of the Inferior Area 6 in the Macaque Monkey.” Journal of Comparative Neurology 251, no. 3: 281–298. 10.1002/cne.902510302. 3021823

[hbm70154-bib-0133] Meyers, E. M. , D. J. Freedman , G. Kreiman , E. K. Miller , and T. Poggio . 2008. “Dynamic Population Coding of Category Information in Inferior Temporal and Prefrontal Cortex.” Journal of Neurophysiology 100, no. 3: 1407–1419. 10.1152/jn.90248.2008. 18562555 PMC2544466

[hbm70154-bib-0136] Mizuguchi, N. , H. Nakata , and K. Kanosue . 2014. “Activity of Right Premotor‐Parietal Regions Dependent Upon Imagined Force Level: An fMRI Study.” Frontiers in Human Neuroscience 8: 810. 10.3389/fnhum.2014.00810. PMC4189331 25339893

[hbm70154-bib-0137] Mizuguchi, N. , M. Sakamoto , T. Muraoka , et al. 2011. “The Modulation of Corticospinal Excitability During Motor Imagery of Actions With Objects.” PLoS One 6, no. 10: e26006. 10.1371/journal.pone.0026006. 22022491 PMC3192791

[hbm70154-bib-0138] Mizuguchi, N. , I. Umehara , H. Nakata , and K. Kanosue . 2013. “Modulation of Corticospinal Excitability Dependent Upon Imagined Force Level.” Experimental Brain Research 230, no. 2: 243–249. 10.1007/s00221-013-3649-3. 23877227

[hbm70154-bib-0139] Momennejad, I. , and J.‐D. Haynes . 2012. “Human Anterior Prefrontal Cortex Encodes the ‘What’ and ‘When’ of Future Intentions.” NeuroImage 61, no. 1: 139–148. 10.1016/j.neuroimage.2012.02.079. 22418393

[hbm70154-bib-0140] Monaco, S. , G. Malfatti , J. C. Culham , L. Cattaneo , and L. Turella . 2020. “Decoding Motor Imagery and Action Planning in the Early Visual Cortex: Overlapping but Distinct Neural Mechanisms.” NeuroImage 218: 116981. 10.1016/j.neuroimage.2020.116981. 32454207

[hbm70154-bib-0142] Myers, N. E. , M. G. Stokes , and A. C. Nobre . 2017. “Prioritizing Information During Working Memory: Beyond Sustained Internal Attention.” Trends in Cognitive Sciences 21, no. 6: 449–461. 10.1016/j.tics.2017.03.010. 28454719 PMC7220802

[hbm70154-bib-0143] Mylopoulos, M. , and E. Pacherie . 2019. “Intentions: The Dynamic Hierarchical Model Revisited.” WIREs Cognitive Science 10, no. 2: e1481. 10.1002/wcs.1481. 30105894

[hbm70154-bib-0144] Nakayama, Y. , T. Yamagata , J. Tanji , and E. Hoshi . 2008. “Transformation of a Virtual Action Plan Into a Motor Plan in the Premotor Cortex.” Journal of Neuroscience 28, no. 41: 10287–10297. 10.1523/JNEUROSCI.2372-08.2008. 18842888 PMC6671018

[hbm70154-bib-0147] Nierhaus, T. , S. Wesolek , D. Pach , C. M. Witt , F. Blankenburg , and T. T. Schmidt . 2023. “Content Representation of Tactile Mental Imagery in Primary Somatosensory Cortex.” Eneuro 10, no. 6: ENEURO.0408‐22.2023. 10.1523/ENEURO.0408-22.2023. PMC10249945 37221090

[hbm70154-bib-0148] Nobre, A. C. , and M. G. Stokes . 2019. “Premembering Experience: A Hierarchy of Time‐Scales for Proactive Attention.” Neuron 104, no. 1: 132–146. 10.1016/j.neuron.2019.08.030. 31600510 PMC6873797

[hbm70154-bib-0149] Norman, K. A. , S. M. Polyn , G. J. Detre , and J. V. Haxby . 2006. “Beyond Mind‐Reading: Multi‐Voxel Pattern Analysis of fMRI Data.” Trends in Cognitive Sciences 10, no. 9: 424–430. 10.1016/j.tics.2006.07.005. 16899397

[hbm70154-bib-0150] Nowak, D. A. , J. Berner , B. Herrnberger , T. Kammer , G. Grön , and C. Schönfeldt‐Lecuona . 2009. “Continuous Theta‐Burst Stimulation Over the Dorsal Premotor Cortex Interferes With Associative Learning During Object Lifting.” Cortex 45, no. 4: 473–482. 10.1016/j.cortex.2007.11.010. 18400218

[hbm70154-bib-0151] Oldfield, R. C. 1971. “The Assessment and Analysis of Handedness: The Edinburgh Inventory.” Neuropsychologia 9, no. 1: 97–113. 10.1016/0028-3932(71)90067-4. 5146491

[hbm70154-bib-0230] Pardo‐Vazquez, J. 2011. “Decision‐Making in the Ventral Premotor Cortex Harbinger of Action.” Frontiers in Integrative Neuroscience 5. 10.3389/fnint.2011.00054. PMC3180590 21991249

[hbm70154-bib-0152] Parr, T. , and K. J. Friston . 2017. “Working Memory, Attention, and Salience in Active Inference.” Scientific Reports 7, no. 1: 14678. 10.1038/s41598-017-15249-0. 29116142 PMC5676961

[hbm70154-bib-0234] Passingham, R. E. 1988. “Premotor Cortex and Preparation for Movement.” Experimental Brain Research 70, no. 3: 590–596. 10.1007/bf00247607. 3384057

[hbm70154-bib-0154] Passingham, R. E. 1989. “Premotor Cortex and the Retrieval of Movement.” Brain, Behavior and Evolution 33, no. 2–3: 189–192. 10.1159/000115927. 2758301

[hbm70154-bib-0155] Passingham, R. E. 1996. “Functional Specialization of the Supplementary Motor Area in Monkeys and Humans.” Advances in Neurology 70: 105–116. 8615192

[hbm70154-bib-0236] Passingham, R. E. , I. Toni , N. Schluter , and M. F. S. Rushworth . 2007. “How Do Visual Instructions Influence the Motor System?” Novartis Foundation Symposium 218 ‐ Sensory Guidance of Movement: 129–146. 10.1002/9780470515563.ch8. 9949819

[hbm70154-bib-0157] Pennock, I. M. L. , T. T. Schmidt , D. Zorbek , and F. Blankenburg . 2021. “Representation of Visual Numerosity Information During Working Memory in Humans: An fMRI Decoding Study.” Human Brain Mapping 42, no. 9: 2778–2789. 10.1002/hbm.25402. 33694232 PMC8127141

[hbm70154-bib-0158] Petit, L. , S. M. Courtney , L. G. Ungerleider , and J. V. Haxby . 1998. “Sustained Activity in the Medial Wall During Working Memory Delays.” Journal of Neuroscience 18, no. 22: 9429–9437. 10.1523/JNEUROSCI.18-22-09429.1998. 9801381 PMC6792871

[hbm70154-bib-0160] Picard, N. , and P. L. Strick . 1996. “Motor Areas of the Medial Wall: A Review of Their Location and Functional Activation.” Cerebral Cortex 6, no. 3: 342–353. 10.1093/cercor/6.3.342. 8670662

[hbm70154-bib-0166] Requin, J. , and A. Riehle . 1995. “Neural Correlates of Partial Transmission of Sensorimotor Information in the Cerebral Cortex.” Acta Psychologica 90, no. 1–3: 81–95. 10.1016/0001-6918(95)00039-W. 8525878

[hbm70154-bib-0167] Ridderinkhof, K. R. , M. Ullsperger , E. A. Crone , and S. Nieuwenhuis . 2004. “The Role of the Medial Frontal Cortex in Cognitive Control.” Science 306, no. 5695: 443–447. 10.1126/science.1100301. 15486290

[hbm70154-bib-0168] Riehle, A. , and J. Requin . 1989. “Monkey Primary Motor and Premotor Cortex: Single‐Cell Activity Related to Prior Information About Direction and Extent of an Intended Movement.” Journal of Neurophysiology 61, no. 3: 534–549. 10.1152/jn.1989.61.3.534. 2709098

[hbm70154-bib-0169] Rizzolatti, G. , L. Fadiga , M. Matelli , et al. 1996. “Localization of Grasp Representations in Humans by PET: 1. Observation Versus Execution.” Experimental Brain Research 111, no. 2: 246–252. 10.1007/BF00227301. 8891654

[hbm70154-bib-0172] Romo, R. , C. D. Brody , A. Hernández , and L. Lemus . 1999. “Neuronal Correlates of Parametric Working Memory in the Prefrontal Cortex.” Nature 399, no. 6735: 470–473. 10.1038/20939. 10365959

[hbm70154-bib-0174] Ruiz, S. , S. Lee , J. L. Dalboni Da Rocha , et al. 2024. “Motor Intentions Decoded From fMRI Signals.” 10.20944/preprints202405.0016.v1. PMC11274965 39061384

[hbm70154-bib-0178] Schmidt, T. T. , and F. Blankenburg . 2019. “The Somatotopy of Mental Tactile Imagery.” Frontiers in Human Neuroscience 13: 10. 10.3389/fnhum.2019.00010. 30833894 PMC6387936

[hbm70154-bib-0180] Schmidt, T. T. , P. Schröder , P. Reinhardt , and F. Blankenburg . 2021. “Rehearsal of Tactile Working Memory: Premotor Cortex Recruits Two Dissociable Neuronal Content Representations.” Human Brain Mapping 42, no. 1: 245–258. 10.1002/hbm.25220. 33009881 PMC7721226

[hbm70154-bib-0181] Schmidt, T. T. , Y. Wu , and F. Blankenburg . 2017. “Content‐Specific Codes of Parametric Vibrotactile Working Memory in Humans.” Journal of Neuroscience 37, no. 40: 9771–9777. 10.1523/JNEUROSCI.1167-17.2017. 28893928 PMC6596612

[hbm70154-bib-0182] Shenoy, K. V. , M. Sahani , and M. M. Churchland . 2013. “Cortical Control of Arm Movements: A Dynamical Systems Perspective.” Annual Review of Neuroscience 36, no. 1: 337–359. 10.1146/annurev-neuro-062111-150509. 23725001

[hbm70154-bib-0185] Soon, C. S. , M. Brass , H.‐J. Heinze , and J.‐D. Haynes . 2008. “Unconscious Determinants of Free Decisions in the Human Brain.” Nature Neuroscience 11, no. 5: 543–545. 10.1038/nn.2112. 18408715

[hbm70154-bib-0186] Soon, C. S. , A. H. He , S. Bode , and J.‐D. Haynes . 2013. “Predicting Free Choices for Abstract Intentions.” Proceedings of the National Academy of Sciences 110, no. 15: 6217–6222. 10.1073/pnas.1212218110. PMC3625266 23509300

[hbm70154-bib-0187] Spitzer, B. , and F. Blankenburg . 2012. “Supramodal Parametric Working Memory Processing in Humans.” Journal of Neuroscience 32, no. 10: 3287–3295. 10.1523/JNEUROSCI.5280-11.2012. 22399750 PMC6621033

[hbm70154-bib-0188] Spitzer, B. , M. Gloel , T. T. Schmidt , and F. Blankenburg . 2014a. “Working Memory Coding of Analog Stimulus Properties in the Human Prefrontal Cortex.” Cerebral Cortex 24, no. 8: 2229–2236. 10.1093/cercor/bht084. 23547134

[hbm70154-bib-0189] Spitzer, B. , D. Goltz , E. Wacker , R. Auksztulewicz , and F. Blankenburg . 2014b. “Maintenance and Manipulation of Somatosensory Information in Ventrolateral Prefrontal Cortex.” Human Brain Mapping 35, no. 5: 2412–2423. 10.1002/hbm.22337. 23913849 PMC6869731

[hbm70154-bib-0190] Spitzer, B. , E. Wacker , and F. Blankenburg . 2010. “Oscillatory Correlates of Vibrotactile Frequency Processing in Human Working Memory.” Journal of Neuroscience 30, no. 12: 4496–4502. 10.1523/JNEUROSCI.6041-09.2010. 20335486 PMC6634519

[hbm70154-bib-0191] Sreenivasan, K. K. , and M. D'Esposito . 2019. “The What, Where and How of Delay Activity.” Nature Reviews Neuroscience 20, no. 8: 466–481. 10.1038/s41583-019-0176-7. 31086326 PMC8801206

[hbm70154-bib-0231] Szczepanski, S. M. , C. S. Konen , and S. Kastner . 2010. “Mechanisms of Spatial Attention Control in Frontal and Parietal Cortex.” Journal of Neuroscience 30, no. 1: 148–160. 10.1523/jneurosci.3862-09.2010. 20053897 PMC2809378

[hbm70154-bib-0194] Tecilla, M. , A. Guerra , L. Rocchi , et al. 2022. “Action Selection and Motor Decision Making: Insights From Transcranial Magnetic Stimulation.” Brain Sciences 12, no. 5: 639. 10.3390/brainsci12050639. 35625025 PMC9139261

[hbm70154-bib-0195] Thickbroom, G. W. , B. A. Phillips , I. Morris , M. L. Byrnes , and F. L. Mastaglia . 1998. “Isometric Force‐Related Activity in Sensorimotor Cortex Measured With Functional MRI.” Experimental Brain Research 121, no. 1: 59–64. 10.1007/s002210050437. 9698191

[hbm70154-bib-0200] Toni, I. , M. Rushworth , and R. Passingham . 2001. “Neural Correlates of Visuomotor Associations.” Experimental Brain Research 141, no. 3: 359–369. 10.1007/s002210100877. 11715080

[hbm70154-bib-0201] Tunik, E. , S. H. Frey , and S. T. Grafton . 2005. “Virtual Lesions of the Anterior Intraparietal Area Disrupt Goal‐Dependent On‐Line Adjustments of Grasp.” Nature Neuroscience 8, no. 4: 505–511. 10.1038/nn1430. 15778711 PMC10719865

[hbm70154-bib-0204] Turella, L. , R. Rumiati , and A. Lingnau . 2020. “Hierarchical Action Encoding Within the Human Brain.” Cerebral Cortex 30, no. 5: 2924–2938. 10.1093/cercor/bhz284. 31942941

[hbm70154-bib-0206] Uluç, I. , T. T. Schmidt , Y. Wu , and F. Blankenburg . 2018. “Content‐Specific Codes of Parametric Auditory Working Memory in Humans.” NeuroImage 183: 254–262. 10.1016/j.neuroimage.2018.08.024. 30107259

[hbm70154-bib-0207] Uluç, I. , L. A. Velenosi , T. T. Schmidt , and F. Blankenburg . 2020. “Parametric Representation of Tactile Numerosity in Working Memory.” Eneuro 7, no. 1: ENEURO.0090‐19.2019. 10.1523/ENEURO.0090-19.2019. PMC7029184 31919053

[hbm70154-bib-0208] Van Ede, F. 2020. “Visual Working Memory and Action: Functional Links and Bi‐Directional Influences.” Visual Cognition 28, no. 5–8: 401–413. 10.1080/13506285.2020.1759744. 33223921 PMC7655037

[hbm70154-bib-0209] Van Ede, F. , and A. C. Nobre . 2023. “Turning Attention Inside Out: How Working Memory Serves Behavior.” Annual Review of Psychology 74, no. 1: 137–165. 10.1146/annurev-psych-021422-041757. 35961038

[hbm70154-bib-0211] Van Nuenen, B. F. L. , J. Kuhtz‐Buschbeck , C. Schulz , B. R. Bloem , and H. R. Siebner . 2012. “Weight‐Specific Anticipatory Coding of Grip Force in Human Dorsal Premotor Cortex.” Journal of Neuroscience 32, no. 15: 5272–5283. 10.1523/JNEUROSCI.5673-11.2012. 22496573 PMC6622097

[hbm70154-bib-0212] Vyas, S. , M. D. Golub , D. Sussillo , and K. V. Shenoy . 2020. “Computation Through Neural Population Dynamics.” Annual Review of Neuroscience 43, no. 1: 249–275. 10.1146/annurev-neuro-092619-094115. PMC7402639 32640928

[hbm70154-bib-0214] Walsh, V. 2003. “A Theory of Magnitude: Common Cortical Metrics of Time, Space and Quantity.” Trends in Cognitive Sciences 7, no. 11: 483–488. 10.1016/j.tics.2003.09.002. 14585444

[hbm70154-bib-0215] Ward, N. S. , S. Bestmann , G. Hartwigsen , et al. 2010. “Low‐Frequency Transcranial Magnetic Stimulation Over Left Dorsal Premotor Cortex Improves the Dynamic Control of Visuospatially Cued Actions.” Journal of Neuroscience 30, no. 27: 9216–9223. 10.1523/JNEUROSCI.4499-09.2010. 20610756 PMC3717513

[hbm70154-bib-0217] Wise, S. P. , D. Boussaoud , P. B. Johnson , and R. Caminiti . 1997. “Premotor and Parietal Cortex: Corticocortical Connectivity and Combinatorial Computations.” Annual Review of Neuroscience 20, no. 1: 25–42. 10.1146/annurev.neuro.20.1.25. 9056706

[hbm70154-bib-0218] Wise, S. P. , G. Di Pellegrino , and D. Boussaoud . 1996. “The Premotor Corte× and Nonstandard Sensorimotor Mapping.” Canadian Journal of Physiology and Pharmacology 74, no. 4: 469–482. 10.1139/cjpp-74-4-469. 8828893

[hbm70154-bib-0220] Wolpert, D. M. , and J. R. Flanagan . 2001. “Motor Prediction.” Current Biology 11, no. 18: R729–R732. 10.1016/S0960-9822(01)00432-8. 11566114

[hbm70154-bib-0222] Wong, A. L. , and A. M. Haith . 2017. “Motor Planning Flexibly Optimizes Performance Under Uncertainty About Task Goals.” Nature Communications 8, no. 1: 14624. 10.1038/ncomms14624. PMC5337982 28256513

[hbm70154-bib-0224] Wu, Y. , I. Uluç , T. T. Schmidt , K. Tertel , E. Kirilina , and F. Blankenburg . 2018. “Overlapping Frontoparietal Networks for Tactile and Visual Parametric Working Memory Representations.” NeuroImage 166: 325–334. 10.1016/j.neuroimage.2017.10.059. 29107771

[hbm70154-bib-0225] Yeşilyurt, B. , K. Uğurbil , and K. Uludağ . 2008. “Dynamics and Nonlinearities of the BOLD Response at Very Short Stimulus Durations.” Magnetic Resonance Imaging 26, no. 7: 853–862. 10.1016/j.mri.2008.01.008. 18479876

[hbm70154-bib-0228] Yokoi, A. , and J. Diedrichsen . 2019. “Neural Organization of Hierarchical Motor Sequence Representations in the Human Neocortex.” Neuron 103, no. 6: 1178–1190.e7. 10.1016/j.neuron.2019.06.017. 31345643

[hbm70154-bib-0226] Zokaei, N. , A. J. Quinn , M. T. Hu , M. Husain , F. Van Ede , and A. C. Nobre . 2021. “Reduced Cortico‐Muscular Beta Coupling in Parkinson's Disease Predicts Motor Impairment.” Brain Communications 3, no. 3: fcab179. 10.1093/braincomms/fcab179. 34514395 PMC8421699

