# Supplementary material for: Decoding Parametric Grip‐Force Anticipation From fMRI Data
Source: Hum Brain Mapp. 2025 Feb 12;46(3):e70154. doi: 10.1002/hbm.70154 (PMC11815324; doi:10.1002/hbm.70154)
Supplement: Supplementary file 1 — DATA S1 Supporting Information. [file HBM-46-e70154-s001.pdf]

Decoding Parametric Grip-Force Anticipation from fMRI-Data: Supplementary materials

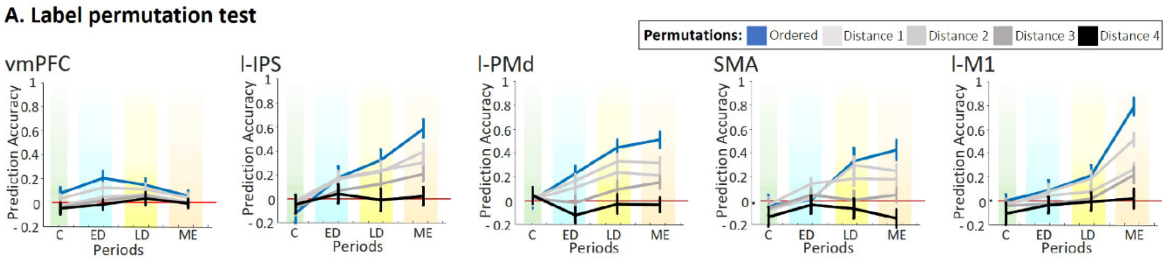

Supplementary Figure S1 A. Results of permutation testing in which the same SVR analysis was performed with permuted labels of the data. Prediction accuracy values were extracted from the peak voxels of the five most representative clusters reported in Table 1 (main text). The time-course represents four prediction accuracy values obtained by averaging prediction accuracy values of twelve time-bins in correspondence of the four time-periods (tested in the main analysis). The divergence of the permutations from the linear order of grip-force levels is expressed as distance in rank order. As expected, the divergence from the original order reduces the performance of the SVR

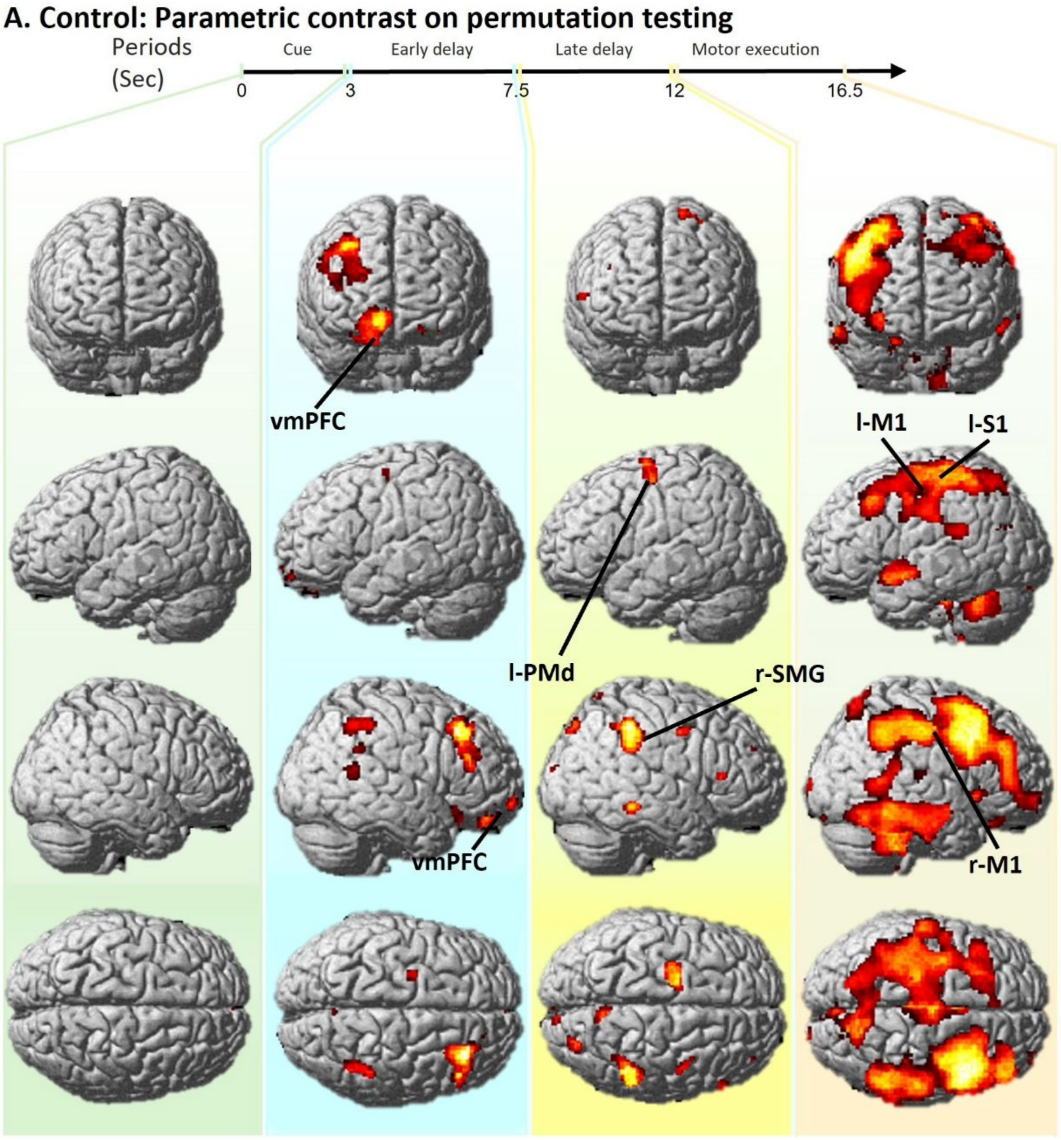

Supplementary Figure S2 A. Results of parametric contrast over prediction accuracy maps from the label permutation tests (see Methods). Parametric contrasts to test for prediction accuracy modulated by the level of order of the permuted labels are presented across the same time periods as in the main analysis (see Figure S1A): cue period (C), early delay period (ED), late delay period (LD) and motor execution period (ME). Above-change prediction accuracy clusters are displayed at p < 0.05, FWE-corrected. No brain region exhibited above-chance decoding during C. As in the main decoding analysis, during ED, a cluster in the vmPFC was revealed and the results of the main analysis were corroborated also for the late delay period, by showing a network including left dorsal premotor cortex (l-PMd), and the right supramarginal gyrus (r-SMG). The left intraparietal sulcus (l-IPS) and supplementary motor areas (SMA) were found at a p < 0.001 uncorrected level. Finally, a t-contrast on the motor execution period revealed bilateral M1 and S1 (fourth column; light red background).

Cluster size	Anatomical Region	Peak MNI coordinates x	y	z	z-score
**Early delay Period**					
780	Ventromedial PFC	8	62	-8	5.57
20	Left frontal pole	-20	62	-16	4.54
1191	Right middle frontal gyrus	28	34	24	5.65
136	Right SMG	44	-48	14	5.23
219	Right angular gyrus	42	-50	48	5.02
52	Left PMd	-24	-8	58	4.93
**Late delay Period**					
341	Left PMd	-24	-10	58	5.57
64	Left SMA	-8	-6	44	4.86
631	Right SMG	50	-38	36	5.79
**Additional cluster in the IPS at p < 0.001**					
356	Left IPS	-30	-56	54	4.10
**Motor execution Period**					
277	Left S1	-60	-18	40	5.03
27045	Left M1	-30	-24	58	6.43

Table S1 Regions that exhibit above-chance prediction accuracy across the cue period, early and late delay periods and motor execution period, revealed by a t-contrast of the parametric permutation testing displayed at p < 0.05, FWE corrected. l-IPS was found at a p < 0. 001 uncorrected level.

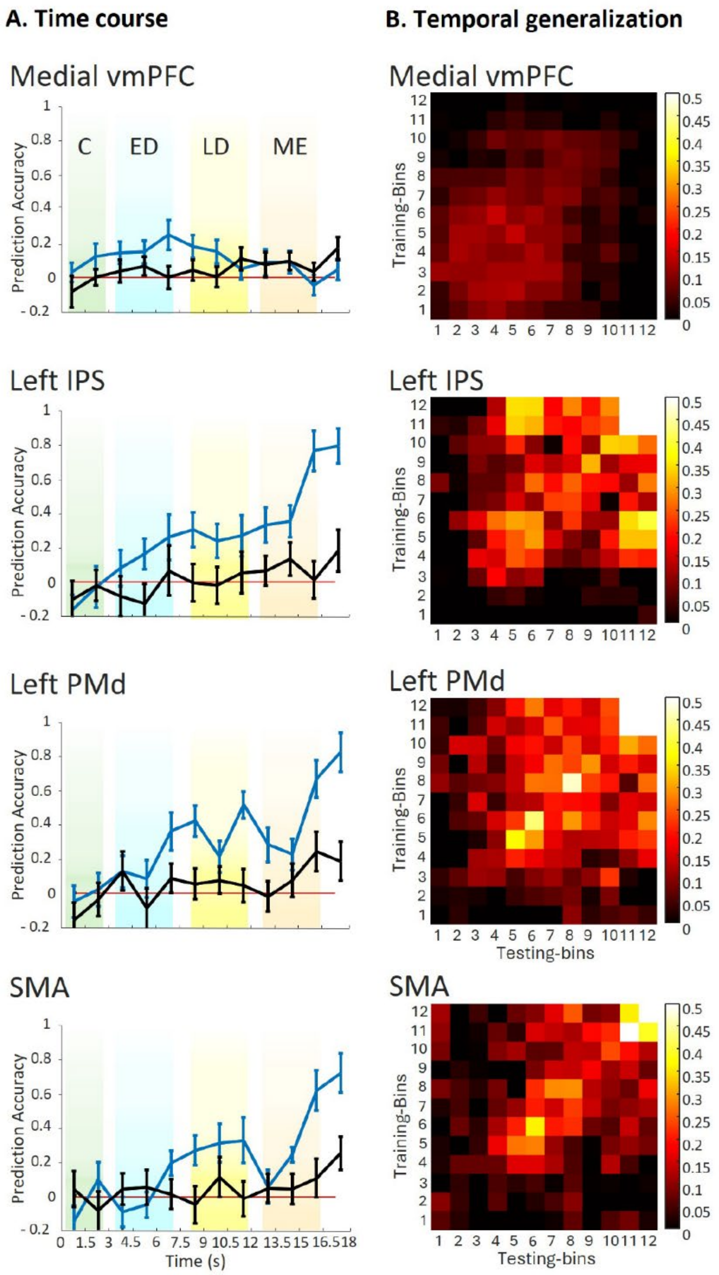

Supplementary Figure S3 A. Time-courses of prediction accuracy values (for all 12 time-bins) relative to the main decoding analysis (displayed in blue) and the control analysis (in black), where the non-memorized grip-force level was decoded. Prediction accuracy values were extracted from the peak voxels of the four most representative clusters reported in Figure 3A, and Table 1 (main text). **B.** Temporal generalization matrices, displaying prediction-accuracy values for the four clusters (with lighter colours indicating higher prediction accuracy values). Prediction-accuracy values were extracted from prediction accuracy maps resulting from the whole-brain searchlight cross-regression decoding, where SVRs were trained on all the time-bins (y-axis) and tested on all the time-bins (x-axis), resulting in 144 cross-regression accuracy maps (t1-t12 x t1-t12).

A. HRF convolved GLM:
Parametric modulation on execution

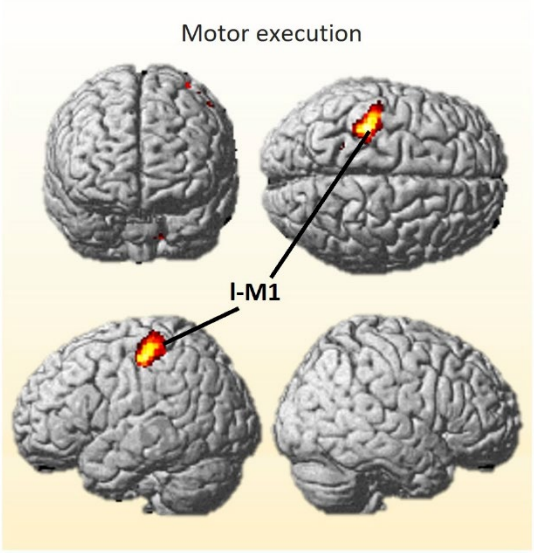

Supplementary Figure S4 A. Results of a one-sample t-test assessing group-level effects on first-level contrasts on beta estimates derived from modelling parametric modulation during motor execution within an HRF-convolved GLM (at the first level analysis). Significant parametric modulation was found in the l-M1 and right cerebellum (at $p < 0.05$ FWE-corrected).

**Motor execution Period**					
881	Left M1	-30	-28	54	6.27
536	Right Cerebellum	22	-50	-20	6.25

Table S2 Regions that exhibit parametric modulation during motor execution, revealed by a t-contrast displayed at $p < 0.05$, FWE corrected.
